# Proteomic analysis may explain differences in *Citrus* × *limon* and *Citrus* × *sinensis* susceptibility to *Trioza erytreae*

**DOI:** 10.1080/15592324.2026.2632509

**Published:** 2026-02-18

**Authors:** T. Magalhães, L. Anjos, S. A. Dandlen, D. M. Power, J. A. Pereira, A. Duarte, Natália Marques

**Affiliations:** a Mediterranean Institute for Agriculture, Environment and Development (MED), Universidade do Algarve, Faro, Portugal; b Mountain Research Center (CIMO), Instituto Politécnico de Bragança, Bragança, Portugal; c Associate Laboratory for Sustainability and Technology (SusTEC), Instituto Politécnico de Bragança, Bragança, Portugal; d Comparative Molecular and Integrative Biology Group, Centre of Marine Sciences (CCMAR), Universidade do Algarve, Faro, Portugal

**Keywords:** Biotic stress, herbivory, HLB, lemon, sweet orange, plant-insect interaction, proteomics, psyllid, sap feeding

## Abstract

The psyllid *Trioza erytreae* is a vector for Huanglongbing, one of the most destructive citrus diseases worldwide. *Citrus* × *limon* is the preferred host, although the mechanisms underlying this preference remain poorly understood. This study compared the responses of *C.* × *limon* ’Eureka’ and *C.* × *sinensis* ‘Valencia’ plants to *T. erytreae* infestation, specifically to nymph feeding. The number of successfully developed nymphs observed in *C.* × *limon* was three times that of ‘Valencia’ plants. The enriched vascular sap proteome of young leaves from infested and control plants was compared using nanoscale liquid chromatography coupled to tandem mass spectrometry. This study identified 48 and 1265 differentially abundant proteins (DAPs) in infested *C.* × *limon* and *C.* × *sinensis*, respectively. There was a marked host-specific response, with little overlap in proteomic features. Common citrus host responses to infestation were the downregulation of ‘Amino sugar and nucleotide sugar metabolism’ and the upregulation of galactose, vitamin B6, and selenocompound metabolisms. The downregulation of photosynthesis-related proteins and the activation of defence-related pathways in *C.* × *sinensis* suggest a robust response, which may explain the low success rate of nymph development on this host. The lower number of DAPs in *C.* × *limon* infested with *T. erytreae*, in comparison to the respective control, may be indicative of a reduced stress response. Further investigation into the identified candidate proteins and pathways is expected to contribute to the elucidation of the interaction between *T. erytreae* and *C.* × *limon*.

## 1 Introduction

Huanglongbing (HLB) is one of the most devastating diseases of the citrus industry and is associated with the presence of the phloem-limited bacterium *Candidatus* Liberibacter spp.. The primary vectors of this disease are the Asian citrus psyllid (*Diaphorina citri* Kuwayama) and the African citrus psyllid (*Trioza erytreae* Del Guercio), although it can also be transmitted by grafting.^1^ Huanglongbing has no viable treatment, so its management is based on vector control and the removal of infested plants.^2^ The Mediterranean basin is one of the most important citrus-growing areas in the world and, so far, has remained free of HLB.^3^ However, a major concern for the citrus industry is the recent introduction of *T. erytreae* into mainland Europe, and its spread along the north and west coasts of the Iberian Peninsula, despite not yet carrying the bacterium.^4^^,^^5^ *Trioza erytreae* thrives on specific hosts, categorized as preferred hosts, common hosts, and occasional hosts.^6^ Preferred hosts are the most attractive to the psyllid, facilitating optimal development. These include lemon plants [*Citrus* × *limon* (L.) Osbeck], white ironwood [*Vepris lanceolata* (Lam.) G. Don] and false horsewood [*Clausena anisata* (Willd.) Hook.f.].^6-8^ Common hosts of intermediate attractiveness to *T. erytreae* include sweet orange [*C.* × *sinensis* (L.) Osbeck], mandarin (*C. reticulata* Blanco) and grapefruit (*C. paradisi* Macfad.).^6^^,^^9^^,^^10^ Trifoliate orange (*C. trifoliata* L.) and cape chestnut (*Calodendrum capense* Thunb.) are considered occasional hosts for the psyllids. These plants have limited attractiveness and provide inadequate support for psyllid growth and development.^6^^,^^11^

Young flushes are critical for *T. erytreae* oviposition and nymphal development.^12^ Oviposition occurs on young shoots, while nymphal development preferentially occurs on the underside of young leaves, resulting in pit gall formation.^13^ The annual cycles of shoot growth and the volatile compounds (limonene, sabinene, and *β*-ocimene) emitted from the leaves of lemon trees are the principal factors explaining the psyllid ´ s preference for this host, which has been identified as the most suitable host.^14^ The oviposition rate of *T. erytreae* is high when the host is lemon.^10^^,^^15^^,^^16^ A comparison of the growth and size of *T. erytreae* nymphs on six Rutaceae hosts showed that nymphs reached their largest size on lemon plants and citrumelo (*C.* × *paradisi* × *C. trifoliata*), followed by sweet orange and *Murraya koenigii* (L.) Spreng plants. In contrast, nymphs developed poorly and were very small on false horsewood and tangelo (*C. reticulata* × *C.* × *paradisi*).^17^

The interaction between sap-sucking insects and their hosts is a complex process that alters the plant´s metabolism and distinguishes susceptible and resistant plant genotypes.^18^ The role of the plant in *T. erytreae* host preference remains underexplored and seems to influence psyllid behavior in terms of attractiveness, oviposition, and nymph development. Previous studies described the impact of plant hosts on insect development in resistant *Pisum sativum* L. (pea) cultivars infested with the phloem-feeding insect *Acyrthosiphon pisum* (Harris). In these instances, the shoot tips contained fewer photosynthesis-related proteins, which hindered insect development.^19^ The response of plants to insect infestation involves the vascular system, which is essential for long- and short-distance transport of nutrients and molecular signals.^20-23^ Adults and nymphs of *Trioza erytreae* feed on phloem sap, with some evidence of xylem consumption.^8^ The vascular system is a site of cross-talk between the psyllid and their host, particularly during the sedentary nymphal stage of development. This interaction modifies local plant responses in such a manner as to render them beneficial to the psyllids.^18^

Phloem sap partitions photoassimilate and is rich in compounds such as ions, metabolites, RNA, immune signals, and proteins, some of which travel long distances in the plant and are part of signaling networks that determine the response of plants to herbivory.^20^^,^^21^^,^^24^ Xylem vessels are responsible for the transportation of water and minerals absorbed by roots from the soil. Additionally, they facilitate the transport of amino acids, carbohydrates, organic acids, and proteins, despite partitioning a significantly lower concentration of proteins than phloem sap.^25-27^ Furthermore, in plants under stress, the xylem is also a route for the jasmonic acid (JA) signaling network.^28^ The findings of Kienow et al.^29^, Koo et al.^30^, and Ruan et al.^31^ indicate that the JA signaling system affects how plants respond to insects. The composition of the apoplast, a free diffusional region made up of the fluid inside the intercellular spaces and the cell wall matrix, provides insight into the mechanisms that underlie the export and import of chemicals by individual cells. Additionally, it sheds light on the intricate interplay between the xylem and phloem compartments.^32^ Knowledge about the composition of vascular sap proteins may reveal the influence of the host plant on nymphal development and contribute to explain the preference of *T. erytreae* for the specific *C.* × *limon* host.

The present study employed proteomics to compare two citrus hosts infested by *T. erytreae*: the preferred host, lemon (*C.* × *limon* ‘Eureka’), and the common host, sweet orange (*C*. × *sinensis* ‘Valencia Midknight Seedless’). The development of *T. erytreae* nymphs on these two hosts was monitored over time. The proteome enriched in vascular sap, derived from the leaf and petiole midribs of young leaves of infested and control plants, was compared to analyze the response of the two citrus host species to *T. erytreae*. The extraction method, which employed centrifugation, resulted in the enrichment of the vascular sap proteome with small amounts of apoplast fluids and the contents of parenchyma and mesophyll cells. The proteome was analyzed using nanoscale liquid chromatography coupled to tandem mass spectrometry (nanoLC-MS/MS). The results of the proteomic study revealed that there was minimal overlap in proteome features between the two citrus hosts infested with psyllids. Furthermore, the proteome of ‘Valencia’ SwO showed the most significant proteome change compared to the respective control.

## 2 Materials and methods

### 2.1 Plant material

The study employed a total of 32 citrus plants, comprising 16 ‘Valencia Midknight Seedless’ sweet orange [*C.* × *sinensis* (L.) Osbeck] (‘Valencia’ SwO) and 16 ‘Eureka’ lemon [*C.* × *limon* (L.) Osbeck] (‘Eureka’ lemon) plants, grafted on ‘Carrizo’ citrange (*C. trifoliata* × *C.* × *sinensis*) rootstock. The citrus plants were purchased from the certified nursery ‘Association of Nurserymen of the District of Coimbra’ (Associação de Viveiristas do Distrito de Coimbra - AVDC) and were all from the same batch. This association is responsible for the propagation of certified material in accordance with European Union legislation.^33^ The plants acquired for this trial had a phytosanitary passport and were categorized as ‘Certified’ category. The plants were two years old and between 0.8 m and 1.0 m in height. The experiment was conducted over a period of four months, from May to July 2021. The plants were cultivated in 5 L tall pots (19 cm in diameter and 25 cm in height) in an artificial potting mix of pine bark and coconut fiber (50:50), fertilized in accordance with standard procedures, and maintained in a climate chamber at 23.5 ± 1 °C, with a relative humidity of 79 ± 5% and a photoperiod of 14:10 h (L:D). Two experimental groups, each comprising eight plants, were established for each citrus species. One group was designated as the control, while the other was subjected to infestation by *T. erytreae*. Three weeks prior to infestation, all 32 plants were pruned in order to induce new shoot growth. The young plants were maintained in controlled conditions throughout the experiment, from the initial pruning stage to the leaf harvest, which occurred between 23 and 25 d after infestation. The number of new shoots exhibited by the plants selected for the experiment was similar. In the infested groups, each plant was infested with 10 *T. erytreae* adults, comprising five males and five females. Prior to leaf harvest, the total number of pit galls on each of the 16 infested plants was counted.

### 2.2 Insect origin and rearing

In 2021, nymphs and adults of *T. erytreae* were collected from pesticide-free lemon orchards in Caracoi (Porto district, Portugal. 41°18 ´ 46.4 ´ ´ N 8°38’09.7’’W). Whenever *T. erytreae* was detected in a new area, the Portuguese official authorities proceeded to test for the presence of *Candidatus* Liberibacter spp.. A random sampling strategy was employed to test for the presence of *Candidatus* Liberibacter spp. in the Caracoi area and throughout Portugal. The tests were based on a polymerase chain reaction (PCR) utilizing primers that were specific for *Candidatus* Liberibacter spp.. The PCR was performed on the insect and the plant samples collected from the field. To date, no samples of *T. erytreae* have tested positive (^34^^,^^35^). Adults of *T. erytreae* were collected using a hand-held aspirator and transferred to conical centrifuge tubes (50 mL). Colonies of *T. erytreae* were established on ‘Eureka’ lemon and ‘Afín Verna 2’ sour orange (*C. aurantium* L.) plants, which were purchased from the certified nursery AVDC with a phytosanitary passport. The infested plants were maintained in acrylic cages (40 × 30 x 43 cm) covered with insect-proof netting, within a climate chamber at 21 ± 1 °C, 50 ± 5% relative humidity, and under a photoperiod of 16:8 h light to dark (L:D). The irrigation of the infested plants used for *T. erytreae* rearing was conducted on a twice-weekly basis, according to the specific needs of the plants. In order to prevent an overabundance of *T. erytreae* in the rearing plant, the insect populations were divided and transferred into new, non-infested plants when necessary, using a handheld aspirator.

### 2.3 Infestation and nymph development

**Figure 1.**
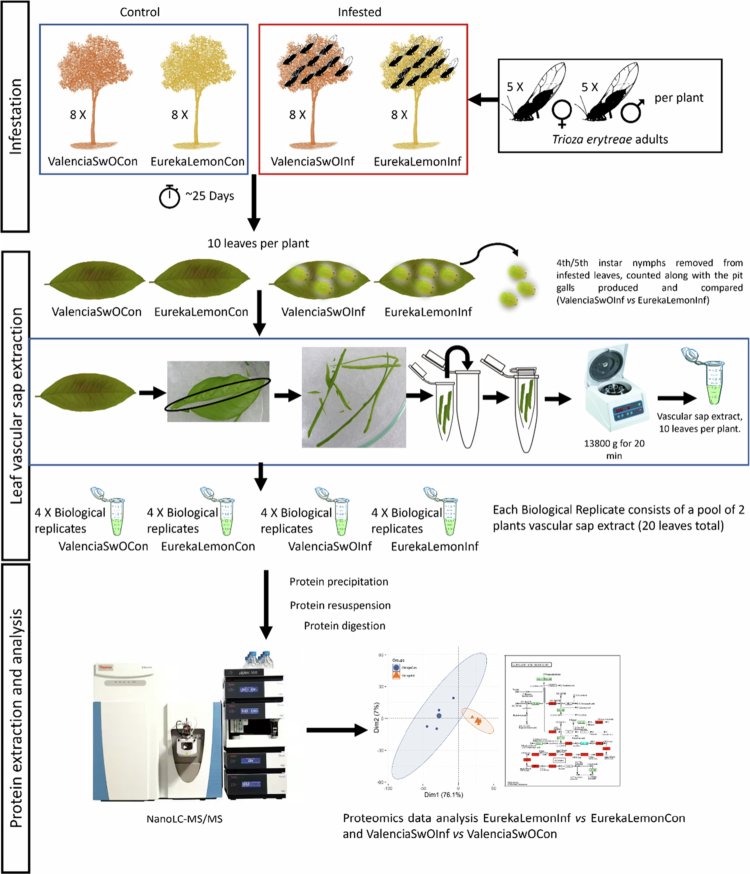
Experimental design and an overview of the workflow showing the main steps: (i) infestation of citrus hosts with *T. erytreae*; ii) counting of nymphs and pit galls; iii) extraction of enriched vascular sap and proteomics analysis. EurekaLemonCon - control ‘Eureka’ lemon plants; EurekaLemonInf - ‘Eureka’ lemon plants infested with *Trioza erytreae*; ValenciaSwOInf—control ‘Valencia’ sweet orange (SwO) plants; ValenciaSwOInf—‘Valencia’ SwO plants infested with *T. erytreae*.

Sexually mature adult psyllids reared in the acrylic cages were used to infest ‘Valencia’ SwO and ‘Eureka’ lemon plants. Ten adult *T. erytreae* specimens, comprising five males and five females, were aspirated from the rearing cages using a hand-held aspirator and collected in a conical centrifuge tube (50 mL). This tube was then used to introduce the psyllids to the netted citrus experimental hosts. A total of eight plants of each species were infested with adult *T. erytreae*, and a further eight plants of each species were used as controls. The experimental groups are hereafter referred to as EurekaLemonInf and ValenciaSwOInf for the infested plants and EurekaLemonCon and ValenciaSwOCon for the control plants (Figure 1). Each plant was isolated within a net that was attached to the tree trunk above the pot to facilitate irrigation and was fixed above the canopy with a wooden frame. The adult psyllids were retained within the net for the duration of the experiment, which spanned 23 to 25 days. The development of the psyllids was monitored over time, as were the numbers of fourth and fifth instar nymphs and the pit galls they formed on the leaves (Figure 1). The total number of pit galls and nymphs was counted immediately before the leaf harvest in both the EurekaLemonInf and ValenciaSwOInf groups.

### 2.4 Citrus enriched vascular sap protein extraction and protein profile analysis

*Trioza erytreae* nymphs were collected from each of the eight infested EurekaLemonInf and ValenciaSwOInf plants when they reached the fourth or fifth instar, which occurred 23–25 days after the infestation. Leaves from the eight infested and eight non-infested plants of each species were collected on the same day and stored at 4 °C until the onset of the protein extraction procedure. Leaves were collected with their petioles intact. The nymphs collected from the infested leaves were counted and removed. Leaves of a similar size and developmental stage as the infested leaves were collected from the non-infested control citrus plants EurekaLemonCon and ValenciaSwOCon. The collection of enriched vascular sap for protein extraction was performed within one hour of the collection of the leaves. Considering the technical constraints associated with the collection of pure vascular sap from young citrus leaves, the Hijaz & Killiny^36^ protocol was adapted. This protocol involves the use of a centrifugal force to facilitate the extraction of enriched vascular sap (Figure 1). The midribs of leaves and petioles of young leaves were isolated and chopped into an appropriate size for a 0.5 mL perforated microtube using a sterilized scalpel. The perforated tube was placed in a 1.5 mL microtube and subjected to centrifugation at 4 °C and 13,800 g (12,000 rpm) for 20 min. The extracted enriched vascular sap was collected at the bottom of the 1.5 mL microtube and stored at −80 °C. The centrifugation method, which employs the use of cut plant material, is primarily effective in the collection of vascular sap and apoplastic fluid. However, this process may also disrupt the cellular structure of younger cells, resulting in an enrichment of the vascular sap with the contents of parenchyma cells, phloem companion cells, and mesophyll cells that were adjacent to the midrib vein. Accordingly, the extracted sap is hereafter designated as ‘enriched vascular sap’.

Acetone, Coomassie blue, trichloroacetic acid (TCA), thiourea, Tris-base, urea and the chemicals required for sodium dodecyl-sulfate polyacrylamide gel electrophoresis (SDS-PAGE) were purchased from Merck KGaA (Darmstadt, Germany), while dithiothreitol (DTT) and hydrochloric acid were purchased from Thermo Fisher Scientific (Eindhoven, The Netherlands). The methodology for total protein extraction was based on a modified approach described by Zadražnik et al.^37^. The enriched vascular sap was extracted from ten leaves of each sampled plant. Four pools were prepared for protein extraction from each experimental group (EurekaLemonInf, ValenciaSwOInf, EurekaLemonCon and ValenciaSwOCon), with each pool comprising two sampled plants (Figure 1). Therefore, four biological replicates were used per each experimental group to ensure the statistical power of the analysis. A total of 100 μL of the extracted protein from each biological replicate was solubilised in 1.25 mL of extraction buffer [10% trichloroacetic acid (TCA), 60 mM dithiothreitol (DTT) in acetone]. The samples were then vortexed and stored at −80 °C overnight. Subsequently, the samples were centrifuged at 14,000 g for 30 min at 4 °C. The pellet was washed three times in 1 mL of wash buffer (60 mM DTT in acetone), followed by a centrifugation at 14,000 g for 5 min at 4 °C. Subsequently, the pellet was left to dry at room temperature and then resuspended in 40 μL of denaturing buffer (7 M urea, 2 M thiourea, 30 mM Tris-HCl, pH 8.5).

The total protein content of the extracted sap was quantified using a Quick Start™ Bradford Protein Assay Kit (Bio-Rad, Hercules, USA) in a Genesys 1Q-S spectrophotometer (Thermo Electron Corporation, Bremen, Germany), with bovine serum albumin (BSA) serving as the standard, in accordance with the manufacturer’s instructions. To evaluate the quality of the protein extracts, 30 μg of total soluble protein from each sample was analyzed by gel electrophoresis in a 12% sodium dodecyl sulfate polyacrylamide gel electrophoresis gel (SDS-PAGE), according to the Laemmli method,^38^ and then stained with Coomassie blue. A total of 50 µg of protein from each sample was subjected to a solid-phase-enhanced sample-preparation (SP3) protocol as described by Hughes et al.^39^, followed by an enzymatic digestion with trypsin/LysC (2 μg) overnight at 37 °C and 1000 rpm. The concentration of the resulting peptides was determined by fluorescence measurement.

### 2.5 Proteomic analysis of citrus enriched vascular sap

#### 2.5.1 Proteomics data acquisition

The citrus enriched vascular sap proteome of the four experimental conditions (EurekaLemonInf, EurekaLemonCon, ValenciaSwOInf and ValenciaSwOCon, *n* = 4 samples per condition) was obtained as described in Osório et al.^40^ Protein identification and quantification were performed by nanoscale liquid chromatography coupled to tandem mass spectrometry (nanoLC-MS/MS) in an *Ultimate* 3000 liquid chromatography system coupled to a Q-Exactive Hybrid Quadrupole-Orbitrap mass spectrometer (Thermo Scientific, Bremen, Germany), with the assistance of an external service provider (Proteomics Scientific Platform of i3S, Ipatimup, Porto, Portugal). Five hundred nanograms of the trypsin/LysC digested samples were loaded onto a trapping cartridge (Acclaim PepMap C18 100 Å, 5 mm × 300 μm i.d., 160454, Thermo Scientific, Bremen, Germany) in a mobile phase of 2% acetonitrile (ACN), 0.1% formic acid (FA) at a flow rate of 10 μL/min. Following a loading period of three minutes, the trap column was switched in-line to a 50 cm × 75 μm inner diameter EASY-Spray column (ES803, PepMap RSLC, C18, 2 μm, Thermo Scientific, Bremen, Germany) at 250 nL/min. The separation was achieved by mixing the mobile phase, comprising A: 0.1% FA and B: 80% ACN, 0.1% FA, with the following gradient: 5 min (2.5% B to 10% B), 120 min (10% B to 30% B), 20 min (30% B to 50% B), 5 min (50% B to 99% B), and 10 min (hold 99% B). Subsequently, the column was equilibrated with 2.5% B for 17 min. The data acquisition process was controlled by Xcalibur 4.0 and Tune 2.9 software (Thermo Scientific, Bremen, Germany). The mass spectrometer was operated in the data-dependent (dd) positive acquisition mode alternating between a full scan (*m*/*z* 380-1580) and subsequent higher-energy collisional dissociation tandem mass spectrometry (HCD MS/MS). This was established for the 10 most intense peaks from a full scan (normalized collision energy of 27%). The electrospray ionization (ESI) spray voltage was at 1.9 kV and the global settings were as follows: lock mass best (*m*/*z* 445.12003), lock mass injection, full MS and chrom peak width at a full width half maximum (FWHM) of 15 s. The full scan settings were as follows: 70 k resolution (*m*/*z* 200), automatic gain control (AGC) target 3 × 10^6^, maximum injection time 120 ms; dd settings: minimum AGC target 8 × 10^3^, intensity threshold 7.3 × 10^4^, charge exclusion: unassigned, 1, 8, >8, peptide matches preferred, exclude isotopes on, and dynamic exclusion 45 s. The MS2 settings were as follows: microscans - 1, resolution - 35 k(*m*/*z* 200), AGC target - 2 × 10^5^, maximum injection time - 110 ms, isolation window - 2.0 *m*/*z*, isolation offset - 0.0 *m*/*z*, dynamic first mass and spectrum data type profile.

#### 2.5.2 Data analysis, protein-label-free quantification and protein identification

The mass spectrometry (MS) raw data were processed using Proteome Discoverer 2.5.0.400 software (Thermo Scientific, Bremen, Germany). Protein identification searches were performed against the UniProt protein sequence database for *C*. × *sinensis* (taxon ID 2711; Release 2020_05; 651,914 sequences) with 44,601 entries and a common contaminant database from MaxQuant (version1.6.2.6, Max Planck Institute of Biochemistry, Munich, Germany). The Sequest HT tandem mass spectrometry peptide database search program was used to identify tryptic peptides, with an ion mass tolerance of 10 ppm for precursors and 0.02 Da for fragmented ions and missing cleavage sites was set as 2. Cysteine carbamidomethylation was defined as a constant modification. Methionine oxidation, asparagine, and glutamine deamidation, peptide *N*-terminus Gln- > pyro-Glut, protein *N*-terminus acetylation, and loss of methionine and Met-loss + Acetyl were defined as variable modifications. Peptide confidence was set to high and the Inferys rescoring node was considered for this analysis. The Percolator processing node was enabled with the following settings: maximum Delta Correlation (deltaCn) 0.05; decoy database search target False Discovery Rate (FDR) 1%; validation based on *q*-value.

Protein-label-free quantification was performed with the Minora feature detector node at the processing step. The following parameters were employed for precursor ion quantification: 1) peptides unique plus razor; 2) precursor abundance was based on intensity; 3) normalization mode was based on the total peptide amount; 4) the minimum number of replicate files was set to 50% in each sample group; 5) the pairwise protein ratio calculation and hypothesis test were based on a *t*-test (background based). The Feature Mapper node from the Proteome Discoverer software was used to generate features from unique peptide-specific peaks within a narrow retention time and mass range (mapping features from different sample files was permitted within a maximum shift of 10 min and 10 ppm of mass tolerance). For the purposes of feature linking and mapping, a signal to noise (S/N) threshold of 5 was employed for the minimum EurekaLemonInf *vs*. EurekaLemonCon comparison and ValenciaSwOInf *vs.* ValenciaSwOCon. The mass spectrometry proteomics data have been deposited in the ProteomeXchange Consortium via the PRIDE^41^ partner repository with the dataset identifier PXD043528 and 10.6019/PXD043528.

#### 2.5.3 Bioinformatics analysis

The fold-change in protein abundance was calculated between the experimental groups EurekaLemonInf and EurekaLemonCon and between ValenciaSwOInf and ValenciaSwOCon. The peptide medians (log2-transformed) between relative protein abundances in the experimental groups were used as the basis for this calculation. The identification of differentially abundant proteins (DAPs) was undertaken. Functional analysis of DAPs was performed using *Arabidopsis thaliana* (L.) Heynh. orthologues of the identified proteins, obtained with the STRING web tool version 11 (https://string-db.org/),^42^ with protein names and definitions obtained from the *Arabidopsis* Information Resource.^43^ In the pursuit of understanding the way in which the citrus plants responds to psyllid infestation, the inherent complexity is mitigated by the superior quality of the protein annotation and functional information derived from *A. thaliana*. The *A. thaliana* database serves as a reference point by its highest number of manually-annotated records. The enrichment analysis was performed using the Kyoto Encyclopedia of Genes and Genomes (KEGG) database (https://www.genome.jp/kegg/)^44^ and the KOBAS web-based tool (http://bioinfo.org/kobas/genelist/).^45^ A hypergeometric test/Fisher’s exact test with a Benjamin and Hochberg^46^ FDR correction method was applied (adjusted *p*-value at < 0.05). Bubble plots and Venn diagrams were created with R software^47^ using the package ggplot2.^48^

### 2.6 Statistical analysis

The mean number of nymphs and pit galls per plant was compared between infested plants, namely EurekaLemonInf and ValenciaSwOInf, using a Student’s *t*-test performed in Rstudio software.^47^ For visualization of data, the package ‘ggstatsplot’^49^ was used in Rstudio, and the standard error of the mean (SEM) is presented. To determine the DAPs enriched in vascular sap between infested and control groups, the following filters were considered: (1) The minimum number of biological samples in which a protein was identified in an experimental group was set to 75% (e.g., 3 out of 4); (2) The presence of at least two unique peptides for protein assignment; and (3) Protein FDR set to a high *q*-value < 0.01. A Student’s *t*-test was performed on the log10 transformed protein abundance with the *p*-value adjusted using a permutation-based correction (with 250 randomizations) (FDR < 0.05). Preprocessing and univariate hypothesis testing were performed in Perseus software version 2.0.7.0. (MaxQuant, Germany).^50^

Principal component analysis (PCA) was performed in Rstudio software^47^ using the built-in ‘prcomp’ function and autoscaled matrices, while the ‘factoextra’ package was used for visualization.^51^ Two types of data were used for the PCA, one comprising all identified proteins in each experimental group, with the application of the filters previously mentioned for the DAP analysis, and the other including only the DAPs. Two permutations of the PCA were performed for the comparisons between the EurekaLemonInf *vs*. EurekaLemonCon groups and the ValenciaSwOInf *vs*. ValenciaSwOCon groups.

## 3 Results

### 3.1 *Trioza erytreae* nymphs developed better on ‘Eureka’ lemon than in ‘Valencia’ sweet orange plants

**Figure 2.**
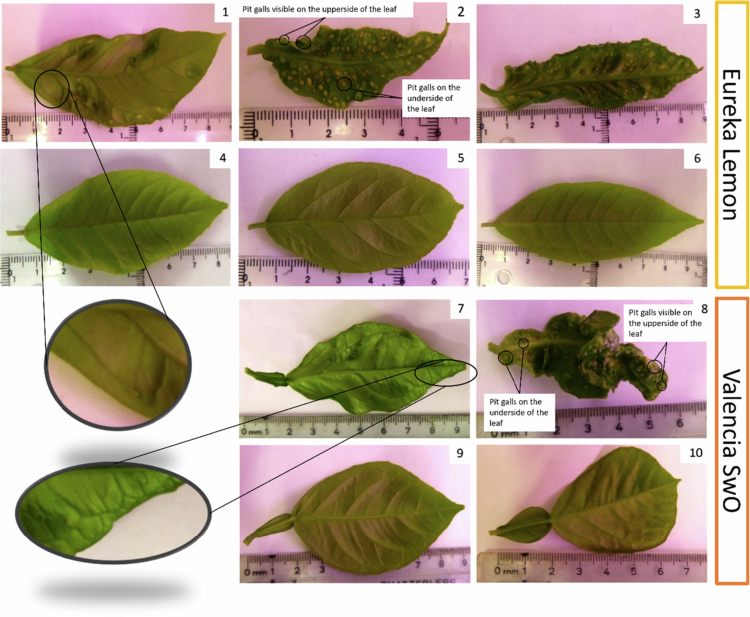
The images show the appearance of ‘Eureka’ lemon and ‘Valencia’ sweet orange (SwO) leaves 23 to 25 days after *Trioza erytreae* infestation. 1, 2 and 3 are infested ‘Eureka’ lemon leaves; 4, 5 and 6 are control ‘Eureka’ lemon leaves; 7 and 8 are infested ‘Valencia’ SwO leaves; 9 and 10 are control ‘Valencia’ SwO leaves. 1- an Infested ‘Eureka’ lemon leaf where 9 nymphs developed, is shown in greater detail in the zoom-in image, which highlights the more pronounced and darker secondary veins; 2- an infested ‘Eureka’ lemon leaf where 51 nymphs developed, is shown in greater detail in the zoom-in image; 3- an Infested ‘Eureka’ lemon leaf where 127 nymphs developed, is shown in greater detail in the zoom-in image (Maximum number of nymphs identified on a ‘Eureka’ lemon leaf); 4, 5 and 6- ‘Eureka’ lemon control leaves of a similar size to 1, 2 and 3, respectively; 7- Infested ‘Valencia’ SwO leaf where 9 nymphs developed, see the the zoom-in. This image highlights the more pronounced and darker secondary veins; 8- This image shows an infested ‘Valencia’ SwO leaf where 58 nymphs developed (the maximum number of nymphs identified on an ‘Valencia’ SwO leaf); 9 and 10- These images show ‘Valencia’ SwO control leaves with similar size to 7 and 8, respectively.

With regard to infestation, the number of new flushes exhibited no significant difference between the different host plants (Welch's *t*-test, *p*-value = 0.15). The mean number of new flushes per plant was 4.63 for ‘Eureka’ lemon and 6.38 for the ‘Valencia’ SwO. The nymphs of *Trioza erytreae* developed to the fourth and fifth instar stage in both the ‘Eureka’ lemon and the ‘Valencia’ SwO plant hosts and induced the formation of pit galls at the feeding sites (see ‘Figure 2.1, 2.2 and 2.3 for ‘Eureka’ lemon; Figure 2.7 and 2.8 for ‘Valencia’ SwO). A more pronounced and darker colouration of the secondary and tertiary leaf vein was observed in a significant proportion of the infested ‘Valencia’ SwO leaves (ValenciaSwOInf, Figure 2, zoom of 2.7), whereas the same symptom was rarely seen in infested ‘Eureka’ lemon leaves (EurekaLemonInf, Figure 2, zoom of 2.1), and not at all in the leaves of control plants. The leaves were deformed when infested with a high number of nymphs (Figure 2.2, 2.3 and 2.8).

**Figure 3.**
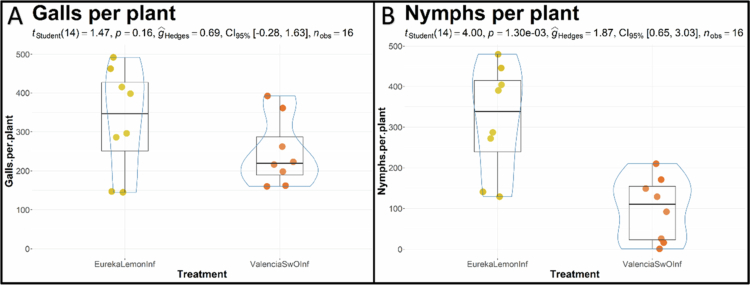
Violin/boxplots illustrate the distribution of pit galls (A) and nymphs (B) per infested plant of ‘Eureka’ lemon (EurekaLemonInf) and ‘Valencia’ sweet orange (ValenciaSwOInf). The statistical analysis applied was a Student’s *t*-test (*t*_Student_) with 14 degrees of freedom, the *p*-value (*p*), the effect size was assessed by Hedge’s *g* (*ĝ*_Hedges_), the confidence interval was set at 95% (CI_95%_), and the number of observations was 16 (*n*_obs_).

The mean number of pit galls per plant from the eight infested ‘Eureka’ lemon plants (EurekaLemonInf) was 330.1 (±47.5 SEM), whereas from the eight infested ‘Valencia’ SwO plants (ValenciaSwOInf) the mean number was 246.8 (±30.8 SEM). The values obtained for these two groups were not significantly different (*p*-value = 0.16) (Figure 3). However, a significant difference was found in the mean number of nymphs (fourth and fifth instar) per host species (*p* < 0.05, Student’s *t*-test). The number of developing nymphs was found to be significantly lower in ValenciaSwOInf hosts, with a mean of 99.3 (±27.6 SEM) nymphs per plant in comparison to EurekaLemonInf, where 318.5 (±47.3 SEM) nymphs developed (Figure 3).

### 3.2 Nymph infestation induces greater proteome changes in ’Valencia’ sweet orange than in ‘Eureka’ lemon

**Figure 4.**
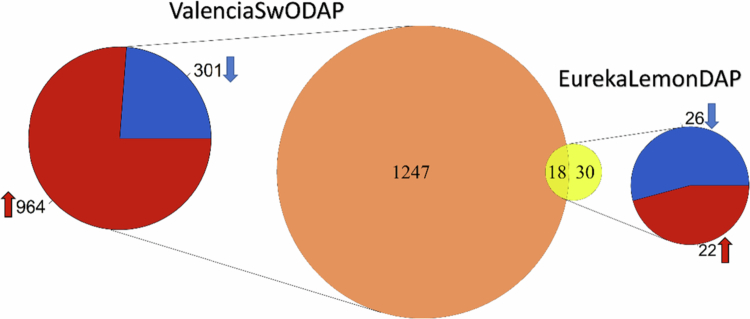
Overview of the differentially abundant proteins (DAPs) in ‘Eureka’ lemon and ‘Valencia’ sweet orange (SwO) plants infested with *T. erytreae* compared to the control plants (ValenciaSwOInf *vs*. ValenciaSwOCon and EurekaLemonInf *vs*. EurekaLemonCon, respectively). The orange circle in the center of the figure shows the 1265 DAPs found in the comparison between infested and control ‘Valencia’ SwO plants (ValenciaSwOInf *vs.* ValenciaSwOCon). Of these, 18 DAPs were common to the comparison between ‘Eureka’ lemon and ValenciaSwO plants, as represented in the intersection with the yellow circle. The smaller yellow circle shows the 48 DAPs found in the comparison between infested and control ‘Eureka’ lemon plants (EurekaLemonInf *vs*. EurekaLemonCon). The pi charts display the distribution of upregulated (red slice) and downregulated (blue slice) proteins in each DAP group. The pi chart on the right-hand side of the figure represents the ‘Eureka’ lemon group (EurekaLemonDAP), and the one on the left-hand side represents the ‘Valencia’ SwO group (ValenciaSwODAP).

The protein extracts from the enriched vascular sap were analyzed by SDS-PAGE, which revealed distinct band profiles between infested and control plants (Supplementary Fig. A). The proteins identified in the enriched vascular sap by nanoLC-MS/MS were profiled and compared between the infested plants and their respective controls to assess the molecular response at the proteome level of ‘Eureka’ lemon and ‘Valencia’ SwO plants infested with *T. erytreae* nymphs. A total of 5050 proteins were identified in the enriched vascular sap. Of these, 33 were classified as contaminants and 1471 had less than two unique peptides and were therefore excluded (Supplementary Table A). A library comprising 3141 and 3370 proteins was generated for ‘Eureka’ lemon and ‘Valencia’ SwO plants, respectively (FDR *q*-value < 0.01) and used for the following comparisons: a) EurekaLemonInf *vs*. EurekaLemonCon; and b) ValenciaSwOInf *vs*. ValenciaSwOCon. A total of 48 DAPs were identified between infested and control ‘Eureka’ lemon plants (EurekaLemonInf *vs.* EurekaLemonCon). Of these, 22 were upregulated and 26 were downregulated (Figure 4 and Supplementary Table B). In contrast, the comparison of the infested and control ‘Valencia’ SwO plants (ValenciaSwOInf *vs*. ValenciaSwOCon) revealed that 964 of the 1265 DAPs were upregulated, while 301 were downregulated (Figure 4 and Supplementary Table B).

In response to *T. erytreae*, a total of 18 DAPs were common to both citrus host species (Figure 4). Of these, nine DAPs were upregulated and four were downregulated in both hosts, while the remaining five were downregulated in ‘Eureka’ lemon plants and upregulated in ‘Valencia’ SwO plants (Table 1). Among the common proteins that were upregulated in infested ‘Eureka’ lemon and ‘Valencia’ SwO plants were methyl esterase 10 (MES10), 3-oxo-2(2′-[Z]-pentenyl) cyclopentane-1-octanoic acid OPC-8:0 CoA ligase (OPLC1), cinnamyl alcohol dehydrogenase 8 (ELI3-2), chlorophyllase 1 (CHL1), raffinose synthase 6 (DIN10 or RS6) and catalase 2 (CAT2) (Table 1).

**Table 1.** Differentially abundant proteins (DAPs) in response to infestation by *Trioza erytreae* nymphs identified in both the ‘Eureka’ lemon and the ‘Valencia’ sweet orange (SwO). The *Arabidopsis thaliana* protein accession codes and protein descriptions were retrieved from the STRING (https://string-db.org/) and TAIR (https://www.arabidopsis.org/) databases.

*C.* × *sinensis* protein accession number (www.uniprot.org/)	*A. thaliana* protein accession number (www.arabidopsis.org/)	EurekaLemonInf Fold Change	ValenciaSwOInf Fold Change	Protein description
A0A067GVT6	CPB (AT1G71790)	0.72	1.33	Subunits of heterodimeric actin filament capping protein Capz superfamily (CPB)
A0A067EE76	MES10 (AT3G50440)	0.65	0.86	Methylesterase 10 (MES10)
A0A067F307	ELI3-2 (AT4G37990)	0.50	0.20	cinnamyl alcohol dehydrogenase 8 (ELI3-2)
A0A067DRF8	AT2G43770	0.46	0.47	Transducin/WD40 repeat-like superfamily protein (AT2G43770)
A0A067G2K8	MTO1 (AT3G01120)	0.39	0.31	Pyridoxal phosphate (PLP)-dependent transferases superfamily protein (MTO1)
A0A067DGV8	CLH1 (AT1G19670)	0.29	0.41	chlorophyllase 1 (CLH1)
A0A067GDP3	DIN10 (AT5G20250)	0.20	0.58	Raffinose synthase family protein (DIN10)
A0A067H294	OPLC1 (AT1G20510)	0.15	0.92	OPC-8:0 CoA ligase1 (OPCL1)
A0A067H2F2	CAT2 (AT4G35090)	0.12	0.65	catalase 2 (CAT2)
A0A067GDZ0	AT5G45910	**(-)** 0.15	0.23	GDSL-like Lipase/Acylhydrolase superfamily protein (AT5G45910)
A0A067FZU2	AT3G11210	**(-)** 0.19	0.33	SGNH hydrolase-type esterase superfamily protein (AT3G11210)
A0A067ESX7	AT3G51680	**(-)** 0.20	0.64	NAD(*P*)-binding Rossmann-fold superfamily protein (AT3G51680)
A0A067DPT0	AT3G05170	**(-)** 0.34	0.26	Phosphoglycerate mutase family protein (AT3G05170)
A0A067CZJ4	AT1G47480	**(-)** 0.38	0.42	alpha/beta-Hydrolases superfamily protein (AT1G47480)
A0A067GVX6	AT2G18570	**(-)** 0.18	**(-)** 0.37	UDP-Glycosyltransferase superfamily protein (AT2G18570)
A0A067D420	CHIA (AT5G24090)	**(-)** 0.351	**(-)** 0.75	chitinase A (CHIA)
A0A067EVR3	APK3 (AT3G03900)	**(-)** 0.46	**(-)** 0.27	adenosine-5'-phosphosulfate (APS) kinase 3 (APK3)
A0A067EPV6	AT5G09880	**(-)** 0.53	**(-)** 0.39	Splicing factor, CC1-like protein (AT5G09880)

**Figure 5.**
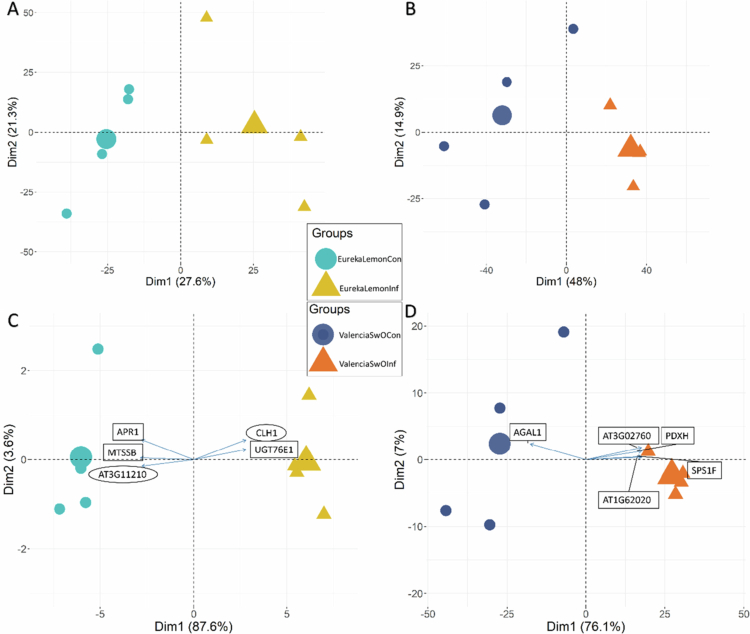
Principal component analysis (PCA) was used to analyze the enriched vascular sap proteomics data of ‘Eureka’ lemon (EurekaLemonInf *vs.* EurekaLemonCon) and ‘Valencia’ sweet orange (SwO) plants (ValenciaSwOInf *vs*. ValenciaSwOCon). The data is presented in the form of a two-dimensional plot, with biological replicates represented by circles (control samples) and triangles (infested samples). The mean value of each replicate group is represented by the largest circle or triangle. The orange color represents the infested ‘Valencia’ sweet orange plants (ValenciaSwOInf), while the dark blue represents the control ‘Valencia’ sweet orange (ValenciaSwOCon). The yellow color represents the infested ‘Eureka’ lemon plants (EurekaLemonInf) while the turquoise represents the ‘Eureka’ lemon control (EurekaLemonCon). A - PCA of ‘Eureka’ lemon plants using all identified proteins; B - PCA of ‘Valencia’ sweet orange plants using all identified proteins; C - PCA of ‘Eureka’ lemon plants using differentially abundant proteins (DAPs); D - PCA of ‘Valencia’ sweet orange plants using DAPs. The arrows indicate the top five greatest weighted variables. The percentages on the axes show their contribution to explain variance.

A PCA was performed using ‘all identified proteins’ from the enriched vascular sap samples of infested and control plants of both citrus host species. The results revealed a clear separation between the two groups along the first principal component axis. The infested plants clustered independently of the control plants in both species, namely in ‘Eureka’ lemon and ‘Valencia’ SwO (Figure 5A and 5B). The first two principal components captured 48.9% and 62.9% of the total data variability for the ‘Eureka’ lemon comparison (Figure 5A) and ‘Valencia’ SwO comparison (Figure 5B), respectively. It may be inferred that the enriched vascular sap proteome was affected by *T. erytreae*.

An additional PCA analysis was conducted using the DAPs from the EurekaLemonInf *vs.* EurekaLemonCon and ValenciaSwOInf *vs*. ValenciaSwOCon comparisons. The results revealed that the first component accounted for 87.6% (Figure 5C) and 76.1% (Figure 5D) of the data variability, respectively. The scatter plot of the DAPs showed a clear separation along the first component axis. The top five proteins with the highest loading values in the two PCA (Supplementary Table C) are displayed in the score scatter plots (Figure 5C and 5D). Two of these proteins, CLH1 and AT3G11210, were classified as ‘common DAPs’ and are highlighted in the ‘Eureka’ lemon comparison (Table 1 and Figure 5C).

The PCA constructed with the DAPs revealed two proteins that were of particular relevance among the five proteins displaying the highest loading values in the EurekaLemonInf *vs.* EurekaLemonCon comparison (Figure 5C). The two proteins are: i) uridine diphosphate glucosyl transferase 76E1 (UGT76E1), which is linked to the wounding response in plants as well as to the formation of JA;^52^ and ii) adenylylsulfate reductase 1 (APR1), which is related to sulfur metabolism.^53^ The proteins with the top five highest loading values in the ValenciaSwOInf *vs*. ValenciaSwOCon comparison (Figure 5D) were linked to biological processes that will be discussed in more detail later. The following proteins were identified: sucrose phosphate synthase 1F (SPS1F), which is related to photosynthesis;^54^ pyridoxine/pyridoxamine 5'-phosphate oxidase 1 (PDXH), which is linked to the metabolism of vitamin B6;^55^ a protein associated with the membrane trafficking coatomer (alpha subunit, AT1G62020);^56^ the protein alpha-galactosidase 1 (AGAL1) and biotin synthetases superfamily (AT3G02760).^57^

### 3.3 Functional analysis

Three clusters from the KEGG pathways were identified as significantly enriched (FDR < 0.05) in DAPs based on comparisons between the EurekaLemonInf *vs*. EurekaLemonCon group and the ValenciaSwOInf *vs*. ValenciaSwOCon group, using *A. thaliana* orthologues (Supplementary Table D). These clusters were classified into three distinct categories: 1) common responses in both groups; 2) opposite regulation of common pathways; and 3) species-specific responses. The common responses included all of the significantly enriched pathways with a common regulation in the ‘Eureka’ lemon and ‘Valencia’ SwO plants. The term ‘opposite regulation of common pathways’ refers to all the significantly enriched pathways with opposite regulation between the two citrus host species. The species-specific responses comprised the KEGG pathways that were significantly and exclusively enriched in one of the citrus host species under study.

#### 3.3.1 *Trioza erytreae*´s nymphs induce common responses from both citrus hosts

**Figure 6.**
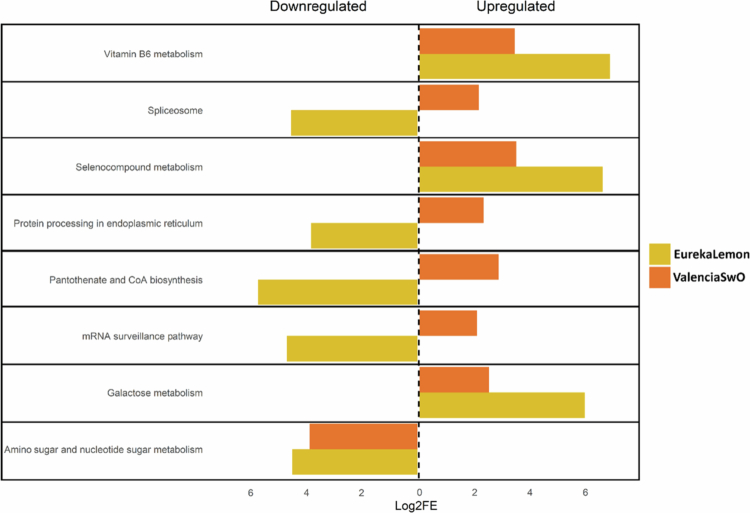
Common KEGG pathways significantly enriched (FDR < 0.05) in the response of ‘Eureka’ lemon and ‘Valencia’ sweet orange (SwO) to *Trioza erytreae* infestation. The enrichment was performed using differentially abundant proteins (DAPs). The bars to the left side of the vertical dashed line represent the downregulated pathways, while those to the right side represent the upregulated pathways. The yellow bars represent the fold enrichment of the EurekaLemonInf *vs*. EurekaLemonCon comparison, while the orange bars represent the fold enrichment of the ValenciaSwOInf *vs*. ValenciaSwOCon comparison. KEGG pathways were considered to be enriched when the false discovery rate (FDR) adjusted *p*-value was below the 0.05 threshold. Fold enrichment was calculated using the *Arabidopsis thaliana* proteome as a reference and log_2_ data transformation.

The KEGG pathways that were common to both citrus hosts may indicate the general response of these two species to psyllid nymph infestation. The four pathways that were commonly and significantly enriched (FDR < 0.05) were ‘Galactose metabolism’, ‘Vitamin B6 metabolism’ and ‘Selenocompound metabolism’, which were upregulated, and ‘Amino sugar and nucleotide sugar metabolism’, which were downregulated (Figure 6).

#### 3.3.2 Protein biosynthesis related pathways with opposite regulation in ‘Eureka’ lemon and ‘Valencia’ sweet orange in response to *Trioza erytreae*

The analysis of the enriched vascular sap of both citrus host species revealed four common enriched KEGG pathways that displayed opposing regulation. These were identified as being downregulated (FDR < 0.05) in ‘Eureka’ lemon and upregulated in ‘Valencia’ SwO plants. The pathways included the ‘spliceosome’, ‘mRNA surveillance pathway’, ‘protein processing in the endoplasmic reticulum’, which are related to protein biosynthesis, and ‘pantothenate and coenzyme-A (CoA) biosynthesis’ (Figure 6).

#### 3.3.3 Two specific ‘Eureka’ lemon pathways in response to *Trioza erytreae*

**Figure 7.**
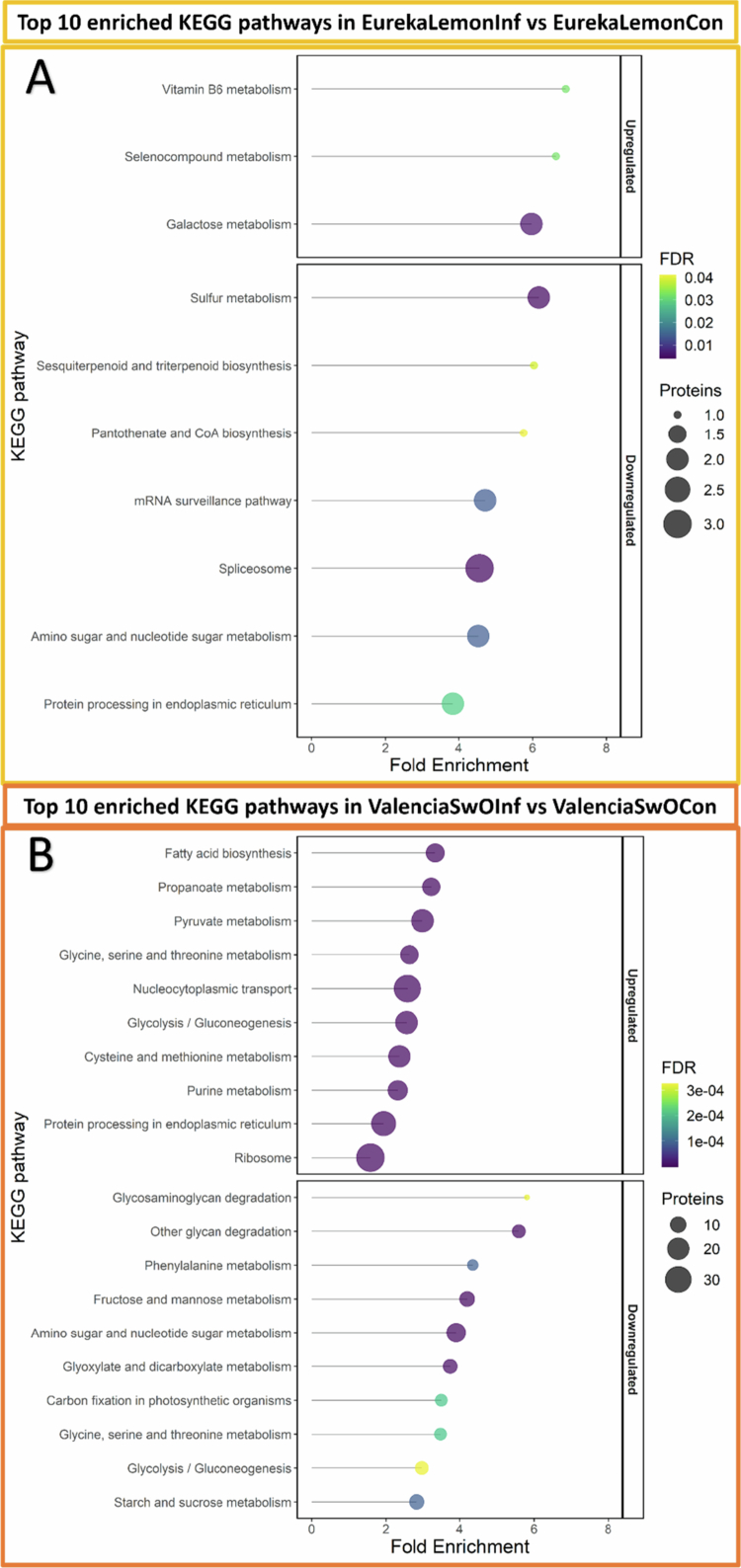
Bubble plot of the top 10 enriched KEGG pathways (based on an FDR-adjusted *p*-value threshold of 0.05) of up and downregulated proteins in infested plants. A - Enriched pathways in the EurekaLemonInf *vs*. EurekaLemonCon comparison. B - Enriched pathways in the ValenciaSwOInf *vs*. ValenciaSwOCon comparison. The size of the bubbles is indicative of the number of proteins present in each pathway, while the color of the bubble represents the FDR-corrected *p*-value and fold enrichment, which were calculated using the *Arabidopsis thaliana* proteome as a reference.

In the EurekaLemonInf group only two KEGG pathways were identified as uniquely enriched: ‘Sulfur metabolism’ and ‘Sesquiterpenoid and triterpenoid biosynthesis’. These pathways were downregulated (Supplementary Table D and Figure 7A).

#### 3.3.4 Bigger and broader adjustment response of ‘Valencia’ sweet orange plants towards *Trioza erytreae* infestation

The ValenciaSwOInf group exhibited a considerably higher number of significantly enriched pathways (86), in comparison to the 10 that were enriched in the EurekaLemonInf group. Of the 86 pathways, 58 were upregulated, and 28 were downregulated (Supplementary Table D). Fifty-one of the upregulated and 27 of the downregulated pathways were uniquely enriched in ValenciaSwOInf. In the ValenciaSwOInf group, the top 10 KEGG pathways enriched with the 964 upregulated proteins were related to biosynthesis, metabolism, and protein synthesis and processing (Figure 7B), while the top 10 KEGG pathways associated with the 301 downregulated proteins were related to metabolism, degradation, and carbon fixation (Figure 7B).

**Figure 8.**
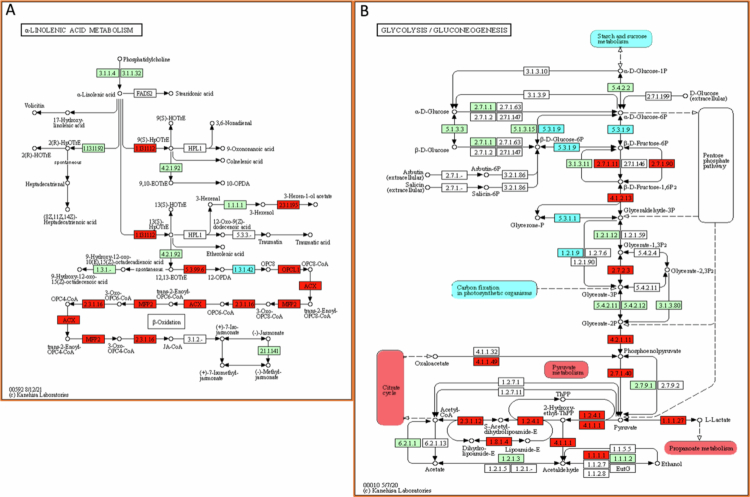
Representative scheme of the enriched KEGG pathways in infested ‘Valencia’ sweet orange plants. KEGG pathway representations are adaptations of the pathway maps available at ‘https://www.genome.jp/kegg/pathway.html The red filled rectangles represent the upregulated proteins and pathways, the blue filled rectangles represent the downregulated proteins and pathways, and the green filled rectangles represent the proteins described for *Arabidopsis thaliana* in this pathway (and that were not differential in this study). A. *α*-Linolenic acid metabolism pathway; B. Glycolysis/Gluconeogenesis pathway.

The proteomics results suggest that there was a significant general metabolic adjustment in the ‘Valencia’ SwO plants. Two plant defense pathways were found to be upregulated, namely ‘α-Linolenic acid metabolism’ (Supplementary Table D and Figure 8A) and ‘Plant-pathogen interaction’. However, these pathways were not among the top 10 pathways identified in Supplementary Table D and Figure 7B. Main upregulated pathways are related to respiration, including ‘Glycolysis and gluconeogenesis’ (Figure 7B and 8B), ‘Pyruvate metabolism’, the ‘Citrate cycle’, and the ‘Propanoate metabolism’ (Supplementary Table D and Figure 8B), as well as ‘Fatty acid biosynthesis’. Pathways that were negatively affected, included ‘Fructose and mannose metabolism’, ‘Glyoxylate and dicarboxylate metabolism’, ‘Starch and sucrose metabolism’ and ‘Carbon fixation in photosynthetic organisms’ (Figure 7B and 8B). *Trioza erytreae* infestation caused changes in amino acid metabolism, as evidenced by the upregulation of the ‘Cysteine and methionine metabolism’ pathway and the downregulation of the ‘Phenylalanine metabolism’ and ‘Glycine, serine, and threonine metabolism’ pathways (Figure 7B).

## 4 Discussion

The high protein levels observed in the samples derived from the leaf and petiole midribs may be attributed to the vascular sap and apoplastic fluid, in addition to the cellular contents of various cell types, which may have been collected during the centrifugation process. Such contents include those of parenchyma cells, phloem companion cells, and mesophyll cells, which were adjacent to the midrib vein of the leaf. The sieve elements are expected to facilitate the transport of a considerable quantity of protein. In fact, studies have described protein synthesis and turnover in the sieve elements, along with the presence of high levels of small RNAs (sRNA) and proteins that have nuclear functions.^58-61^ The process of centrifugation to enrich our samples with vascular sap was previously described by Franco et al.^62^ as a method that can also extract proteins from nuclei.

## 4.1 *Trioza erytreae* nymphs developed better in ‘Eureka’ lemon in comparison to ‘Valencia’ sweet orange

The sexually mature adult *T. erytreae* individuals used in this experiment were randomly reared on sour orange and lemon plants. The use of these plants for psyllid rearing may induce a preference bias for the lemon tree as a host. A preference bias was observed in *D. citri.*^63^ However, Moran^15^ reported that the plant species used for rearing *T. erytreae* (even after two generations) had no effect on host preference. Furthermore, it has been reported that the intensity of flushing affects the development of *T. erytreae.*^64^ However, the present study revealed no statistically significant differences in the number of new flushes between the two citrus host species assayed, thereby suggesting that this trait is unlikely to serve as a differentiating factor.

It was anticipated that the number of nymphs per citrus host would be comparable, given that each citrus host was exposed to the same number of female and male adult insects. Additionally, a single *T. erytreae* nymph is responsible for the formation of a single pit gall, which typically occurs within the initial days following oviposition (early instars).^65^ The number of pit galls formed on the ‘Eureka’ lemon and the ‘Valencia’ SwO hosts was comparable. However, the observation of a reduced number of nymphs developing in ‘Valencia’ SwO plants, in conjunction with the presence of numerous leaves exhibiting a darker color in the secondary and tertiary leaf veins, suggests that the ‘Valencia’ SwO host plant response may have had a detrimental impact on nymph development. The response of the two citrus host species to psyllid infestation was expected to be revealed in the composition of the enriched vascular sap, which transports nutrients and metabolites in response to biotic stress, including molecules of the immune response.^23^

### 4.2 Nymph infestation induces greater proteome changes in ‘Valencia’ sweet orange than in ‘Eureka’ lemon

Of the nine common proteins that were upregulated in both citrus hosts (Table 1), of particular note was MES10 and OPLC1, which are both related to JA metabolism and the ‘JA signaling pathway’. This pathway represents one of the plant responses to insect infestation.^29-31^ Additionally, other upregulated proteins were associated with the response to herbivory and bacteria, respectively, ELI3-2, encoded by the gene *AT4G37990*, and CHL1.^66^^,^^67^ The proteins DIN10 and CAT2 are involved in the response of plants to oxidative stress.^68^^,^^69^ The upregulation of CAT2 and phosphatase 2 A (PP2A) in the citrus plants may indicate that these proteins play a pivotal role in the response to the psyllid infestation. Indeed, wild-type *A. thaliana* exhibited a reduction in the population of phloem-feeding insects in comparison to mutant plants lacking one of the aforementioned proteins.^70^ Among the set of downregulated proteins, chitinase A (CHIA), was identified as one of the four proteins that were downregulated in both citrus hosts (Table 1). This protein has previously been described to be a common constituent of phloem and xylem sap.^32^ Furthermore, diverse chitinases were modified in response to insect infestation in tea plants (*Camellia sinensis* (L.) O. Kuntze).^71^

The higher number of DAPs in the ‘Valencia’ SwO in comparison with the ‘Eureka’ lemon plants (Figure 4) indicates a higher adjustment of the former´s proteome to *T. erytreae* nymph infestation. It is noteworthy that exposure to the bacteria that causes HLB disease revealed that the ‘Lisbon’ lemon exhibited a lower number of DAPs than the ‘Washington Navel’ SwO.^72^ The reduced reprogramming of the defense response may explain why lemon plants are the preferred host of *T. erytreae* and nymphs develop more successfully. Indeed, it has been demonstrated that nymphs developed in lemon plants exhibited a larger size,^6^^,^^17^ and that the psyllid had a higher intensity of settlement, probing, and feeding (Benhadi -Marín et al., 2021). Conversely, the interaction between the psyllid and the lemon plant may result in the activation of additional responses that are not covered in a proteomic approach. One potential avenue for further investigation would be to study the transcription of regulatory genes that impact host vulnerability. Another mechanism to be studied may be the induction of the salicylic acid (SA) pathway, which is commonly induced by aphids and sap-sucking insects. This pathway prevents the plant from fully activating the JA and ethylene plant defense pathways.^18^ Nevertheless, this pathway was not identified in ‘Eureka’ lemon plants in the present study.

### 4.3 Functional analysis

#### 4.3.1 *Trioza erytreae*´s nymphs induce common responses in both citrus host species

In response to the infestation of *T. erytreae*, both citrus host species exhibited an upregulation of proteins involved in ‘Galactose metabolism’ and ‘Vitamin B6 metabolism’ pathways (Figure 6). The only protein from the ‘Galactose metabolism’ pathway that was upregulated in both species was raffinose synthase 6 (DIN10 or RS6) (Supplementary Tables B and D). In general, the response of plants to stress involves raffinose synthases^73^ and the ‘Galactose metabolism’ pathway has been related to ascorbate biosynthesis,^74^ which scavenges reactive oxygen species (ROS) and so protects plant cells.^75^ Additionally, both citrus host species exhibited an upregulation of proteins related to vitamin B6 synthesis (Figure 6). In particular, aldolase-type triosephosphate isomerase (TIM) barrel family protein (RSR4) was upregulated in the ‘Eureka’ lemon, whereas in ‘Valencia’ SwO, bifunctional pyridoxine (pyridoxamine) 5’-phosphate oxidase (PDXH) and pyridoxamine 5-phosphate oxidase (AT2G46580) were found to be upregulated (Supplementary Tables B and D). Vitamin B6 has been described as having antioxidant properties and to regulate redox balance during the defense response of tobacco (*Nicotiana tabacum* L.) to bacteria.^76^ A deficiency of vitamin B6 has been shown to render plants more susceptible to biotic stress.^77^^,^^78^ Moreover, the regulation of the sink and source dynamics in plants may also explain the enrichments of both pathways. This phenomenon may be attributed to the fact that insects that feed on the phloem can act as additional sinks for the plant while infesting it.^79^ The phloem-feeding psyllid, *D. citri*, has been described to ingest galactose.^80^^,^^81^ We hypothesize that *T. erytreae* might act as a galactose sink, which would explain the upregulation of proteins from the ‘Galactose metabolism’ pathway. Additionally, B-complex vitamins are essential for insect nutrition due to their inability to synthesize these compounds de novo.^82^ The downregulation of the ‘Amino sugar and nucleotide sugar metabolism’ pathway in both citrus host species (Figure 6) is consistent with the description of an adjustment in amino acid balance in plants affected by phloem feeders.^83^ Indeed, a distinct amino acid profile was observed in mandarin plants in response to infestation by *D. citri.*^84^

#### 4.3.2 Protein biosynthesis related pathways with opposite regulation in ‘Eureka’ lemon and ‘Valencia’ sweet orange in response to *Trioza erytreae*

The upregulation of proteins from the ‘spliceosome’, ‘mRNA surveillance pathway’, ‘protein processing in endoplasmic reticulum’, ‘nucleocytoplasmic transport’, ‘ribosome’ and ‘pantothenate and coenzyme-A (CoA)’ pathways in ‘Valencia’ SwO plants is indicative of an increased flux of proteins and possible enhanced protein synthesis rate (Figure 6 and 7B). Pathways related to protein biosynthesis were identified as being enriched in maize (*Zea mays* L.), and lima bean (*Phaseolus lunatus* L.) in response to herbivory and other biotic stressors.^85^^,^^86^ Notably, none of these pathways were upregulated in ‘Eureka’ lemon plants. Furthermore, the ‘mRNA surveillance pathway’ and the endoplasmic reticulum (ER) associated degradation system (ERAD) were significantly different between the two citrus host species. The proteins in question were downregulated in EurekaLemonInf and upregulated in ValenciaSwOInf (Figure 6 and Supplementary Fig. B). As the ERAD addresses misfolded and unfolded polypeptides,^87^ an increase in protein synthesis is typically associated with the plant´s response to biotic stress and the synthesis of stress-related proteins.^88^

In summary, the different proteomic fingerprints of the common functional pathways with opposing regulation identified in the enriched vascular sap proteome appear to be related with the suitability of the two citrus host species to *T. erytreae*. It is noteworthy that the ‘Eureka’ lemon, which is the most suitable host, and ‘Valencia’ SwO plants seem to exhibit dissimilar responses to the psyllid infestation.

#### 4.3.3 Two specific downregulated ‘Eureka’ lemon pathways in response to *Trioza erytreae*

The proteins that were uniquely enriched and downregulated in EurekaLemonInf belong to the ‘Sulfur metabolism’ and the ‘Sesquiterpenoid and triterpenoid biosynthesis’ pathways (Supplementary Table D and Figure 7A). Interestingly, the ‘Sulfur metabolism’ pathway was enriched in SwO and two additional Rutaceae hosts infested with *D. citri*, namely *Murraya paniculata* (L.) Jack and *C. japonica* Thunb.. However, this pathway was not enriched in the more attractive mandarin host.^89^ The downregulation of proteins from the ‘Sulfur metabolism’ pathway may explain the preference of *T. erytreae* nymphs for lemon hosts. Sulfur metabolism plays a pivotal role in plant growth, survival, and defense.^90^ In addition, the downregulation of proteins from the ‘Sulfur metabolism’ pathway may be related to the upregulation of proteins from the ‘Selenocompound (*Se*) metabolism’ pathway (Figure 7A), as *Se* and sulfur isolog compounds compete in biochemical processes.^91^^,^^92^ Terpene synthase 21 (TPS21) from the ‘Sesquiterpenoid and triterpenoid biosynthesis’ pathway was downregulated in EurekaLemonInf and upregulated in ValenciaSwOInf (Figure 7A and Supplementary Table B). This enzyme plays a pivotal role in the formation of sesquiterpenes.^93^ In mandarin plants infested by the psyllid *D. citri*, an increase in the leaf volatile sesquiterpene was observed.^94^ These findings also suggest that the detected compounds were not derived solely from the vascular sap.

In conclusion, the limited number of significantly and uniquely enriched pathways in infested ‘Eureka’ lemon seems to indicate that their defense mechanism is very distinct from that of ‘Valencia’ SwO. This may also explain the higher survival rate of *T. erytreae* nymphs in ‘Eureka’ lemon compared to ‘Valencia’ SwO (Figure 3). It is plausible that additional defensive mechanisms may have been triggered in the ‘Eureka’ lemon that were not identified in enriched vascular sap using proteomics.

#### 4.3.4 Bigger and broader adjustment of ‘Valencia’ sweet orange plants towards *Trioza erytreae* infestation

Of particular interest among the pathways that were uniquely enriched and upregulated in ValenciaSwOInf was the ‘fatty acid biosynthesis’ pathway (Figure 7B). This pathway is associated with the synthesis of structural barriers to the external environment and the adjustment of cell membrane fluidity, which is a useful stress response.^95^ Furthermore, the ‘fatty acid biosynthesis’ and ‘α-Linolenic acid metabolism’ pathways (Supplementary Table D and Figure 8A) were upregulated. Proteins involved in the ‘α-Linolenic acid metabolism’ pathway are associated with the synthesis of JA (Figure 8A), a general pattern activated in plants exposed to insect herbivory.^31^^,^^95^ For example, ´ Valencia ´ SwO variety exposed to *D. citri* exhibited increased jasmonic acid (JA) levels.^96^ The same insect-host system also showed upregulated lipoxygenase (LOX), allene oxide cyclase (AOC) and oxophytodienoate-reductase 3 (OPR3) gene transcripts, which are affected to the JA signaling pathway.^97^^,^^98^ Both LOX2 and AOC3 were upregulated in *T. erytreae* infested ‘Valencia’ SwO plants (Supplementary Table B) and these proteins were previously reported as common constituents of the phloem sap defense response.^99^ The current findings suggest that the JA signaling pathway is triggered in SwO in response to the two psyllids.

The upregulation of respiration metabolism-related pathways, including ‘Pyruvate metabolism’, ‘Citrate cycle’, ‘Glycolysis/Gluconeogenesis’, the ‘Propanoate metabolism’, and the concomitant downregulation of photosynthesis-related pathways, such as the ‘Carbon fixation in photosynthetic organisms’ (Supplementary Table D, Figure 7B and 8B) represents a general pattern of plant response to stress.^100^

Concerning the downregulation of the ‘Carbon fixation in photosynthetic organisms’ pathway, it is represented by the alanine aminotransferase 2 (ALAAT2), aspartate aminotransferase 3 (ASP3), triosephosphate isomerase (TPI), and two fructose-bisphosphate aldolases (FBA2 and FBA5) (Supplementary Tables B and D). Similarly, SwO ’Madam Vinous’ and tomato (*Solanum lycopersicum* L.) plants showed a downregulation of photosynthesis-related pathways and TPI proteins, respectively, in response to sap-feeding insects.^101^^,^^102^ The observed phenotypic alterations in the secondary and tertiary veins of ‘Valencia’ SwO leaves may be attributed to alterations in protein pathways, the disassembly of chloroplasts, and the reallocation of nutrients.

A considerable number of proteins related to the ‘Photorespiration’ pathway module, such as the alanine:glyoxylate aminotransferase (AGT) and serine hydroxymethyltransferase 1 and 3 (SHM1 and SHM3), were negatively regulated (Supplementary Tables B and D, and Supplementary Fig. C). Given that photorespiration affects several primary metabolic pathways, it is plausible that these proteins exert an influence on the growth and development of the ‘Valencia’ SwO host. On the opposite, the exposure to phloem-feeding insects led to the high expression of photorespiration-linked pepper (*Capsicum annuum* L.) proteins,^103^ whereas proteins SHM1 and SHM3 of olive (*Olea europaea* L.) were downregulated.^104^ Therefore, it may be concluded that the type of stress experienced and the specific host are determining factors in the regulation of this pathway.

The present study identified a wide range of proteins from the ‘plant-pathogen interaction’ pathway (outside of the top ten KEGGs) that were upregulated in ‘Valencia’ SwO plants (Supplementary Tables B and D and Supplementary Fig. C). To the best of our knowledge, this pathway has only been observed to be upregulated in citrus plants in response to fungi^105^ and bacteria,^106^^,^^107^ but not when exposed to a psyllid. In particular, the hypersensitive response seems to be activated in ‘Valencia’ SwO plants by the upregulation of the protein kinases 3 and 4 (MPK3 and MPK4), cysteine- and histidine-rich domain-containing protein RAR1 (PBS2 or PPHB susceptible 2), and calcium-dependent protein kinases 6 and 9 (CPK6 and CPK9) (Supplementary Tables B and D and Supplementary Fig. C). Although bacteria and fungi are commonly identified as the primary agents responsible for the hypersensitive response, recent research suggest that insects may also elicit this response.^108^ Calcium-related protein kinases (CPK6 and CPK9), which serve as sensors of wounding and feeding,^97^ have been identified in ´ Valencia ´ SwO in response to *D. citri*, being described as phloem sap defense proteins.^99^^,^^109^ According to Sun et al.^98^, the ‘Succari’ SwO response to *D. citri* resulted in the downregulation of CPK9 transcripts, a response that was not observed in ´ Valencia’ SwO. Proteins of the ‘Salicylic acid (SA) pathway’, which are frequently upregulated during a plant defensive response, were not identified in the responses of the two citrus host species to the psyllid. In contrast, the ‘Succari’ SwO responded to *D. citri* by upregulating gene transcripts associated with the SA pathway.^98^

Overall, the fact that more enriched pathways were found in the infested ‘Valencia’ SwO plants than in the infested ‘Eureka’ lemon plants indicates that the former exhibited a more pronounced response to the *T. erytreae* infestation. The response was characterized by metabolic, protein synthesis, and defense adjustments. Furthermore, the present results seem to indicate that the ‘Valencia’ SwO triggers the hypersensitive response. The collective effect of these proteomic modifications may hamper the development of *T. erytreae* nymphs.

## 5 Conclusion

This study provides an in-depth analysis of the enriched vascular sap proteome of ‘Eureka’ lemon and ‘Valencia’ SwO when infested with *T. erytreae* nymphs. The results reveal that nymphs caused a significant modification of the proteome of the enriched vascular sap of young leaves. Upon exposure to *T. erytreae*, the ‘Valencia’ SwO showed the most noticeable alterations, which included upregulation of proteins related to respiration, protein biosynthesis, and stress-specific responses, such as the hypersensitive response, antioxidant proteins, and JA signaling pathways. The downregulation of proteins associated with photosynthesis, photorespiration, and carbohydrate synthesis in the enriched vascular sap of ‘Valencia’ SwO suggests that these proteins are part of a defensive response, as such responses are commonly observed in plants infested by sap-feeding insects. Furthermore, proteins that form structural barriers and alter the fluidity of cell membranes were found to be upregulated. These modifications are considered to be typical responses to biotic stress and may be indicative of a defensive mechanism that renders this host less suitable for the growth of *T. erytreae*. The proteomic response of ‘Eureka’ lemon to *T. erytreae* infestation was less pronounced than that observed in the ‘Valencia’ SwO response. Of particular note was the downregulation of sulfur metabolism, which plays a pivotal role in plant defense. In conclusion, the proteomic analysis of the enriched vascular sap of the two citrus host species revealed distinct responses to psyllid infestation. The present results prompt further investigation into alternative defense responses that may have been activated and not captured in the proteomic study. The current findings identify promising new areas for research and emphasize the need for further studies into the cross-talk between *T. erytreae* and citrus species, to define the factors that affect the choice of host plant by the psyllids.

## Supplementary Material

Supplementary_materials Supplementary_materials

Table_D_KEGG pathway enrichment analysis for the response of Eureka lemon and Valencia SwO to Trioza erytreae .xlsx Table_D_KEGG pathway enrichment analysis for the response of Eureka lemon and Valencia SwO to Trioza erytreae .xlsx

Table_A_Protein library resulting from the nano LC MSMS analysis of the EurekaLemonInf EurekaLemonCon .xlsx Table_A_Protein library resulting from the nano LC MSMS analysis of the EurekaLemonInf EurekaLemonCon .xlsx

Table_C_Loading values of the differentially abundant proteins DAPs related to the first component of the principal com.xlsx Table_C_Loading values of the differentially abundant proteins DAPs related to the first component of the principal com.xlsx

Figure_B_Representation of the enriched Protein Processing in the Endoplasmic reticulum pathway of EurekaLemonInf and ValenciaSwOInf plants.docx Figure_B_Representation of the enriched Protein Processing in the Endoplasmic reticulum pathway of EurekaLemonInf and ValenciaSwOInf plants.docx

Table_B_Differentially abundant proteins DAPs found in lemon and orange plants in response to Trioza erytreae infestatio.xlsx Table_B_Differentially abundant proteins DAPs found in lemon and orange plants in response to Trioza erytreae infestatio.xlsx

Figure_C_Representation of the enriched pathways of PlantPathogen Interactions Glyoxylate and dicarboxylate metabolism.docx Figure_C_Representation of the enriched pathways of PlantPathogen Interactions Glyoxylate and dicarboxylate metabolism.docx

Figure_A_SDS PAGE Gel images of the EurekaLemonInf EurekaLemonCon ValenciaSwOInf and ValenciaSwOCon enriched vascular sap proteome profiles.docx Figure_A_SDS PAGE Gel images of the EurekaLemonInf EurekaLemonCon ValenciaSwOInf and ValenciaSwOCon enriched vascular sap proteome profiles.docx

## Data Availability

The mass spectrometry proteomics data have been deposited to the ProteomeXchange Consortium via the PRIDE^41^ partner repository with the data set identifier PXD043528 and 10.6019/PXD043528. All other data supporting the findings of this study are available in the paper and its online supplementary data.

## Abbreviations

ACN: acetonitrile
CoA: coenzyme A
DAPs: differentially abundant proteins
ER: endoplasmic reticulum
ERAD: endoplasmic reticulum associated degradation system
EurekaLemonCon: ‘Eureka’ Lemon control
EurekaLemonInf: infested ‘Eureka’ lemon
FA: formic acid
FDR: false discovery rate
HLB: huanglongbing
JA: jasmonic acid
KEGG: Kyoto Encyclopedia of Genes and Genomes
nanoLC-MS/MS: nanoscale liquid chromatography coupled to tandem mass spectrometry
ROS: reactive oxygen species
SwO: sweet orange, *C.* × *sinensis*
ValenciaSwOCon: ‘Valencia’ sweet orange control
ValenciaSwOInf: infested ‘Valencia’ sweet orange
