# Supplementary material for: Proteomic analysis may explain differences in Citrus × limon and Citrus × sinensis susceptibility to Trioza erytreae
Source: Plant Signal Behav. 2026 Feb 18;21(1):2632509. doi: 10.1080/15592324.2026.2632509 (PMC12928643; doi:10.1080/15592324.2026.2632509)
Supplement: Supplementary_materials [file KPSB_A_2632509_SM5812.docx]

## Appendix. Supplementary materials

Figure A. SDS-PAGE Gel images of the EurekaLemonInf, EurekaLemonCon, ValenciaSwOInf and ValenciaSwOCon enriched vascular sap proteome profiles.

Figure B. Representation of the enriched Protein Processing in the Endoplasmic reticulum pathway of EurekaLemonInf and ValenciaSwOInf plants.

Figure C. Representation of the enriched pathways of “Plant-Pathogen Interactions”, “Glyoxylate and dicarboxylate metabolism”, “Pyruvate metabolism”, “Citrate cycle (TCA cycle)” in ValenciaSwOInf plants.

Table A. Protein library resulting from the nano LC-MS/MS analysis of the EurekaLemonInf, EurekaLemonCon, ValenciaSwOInf and ValenciaSwOCon samples.

Table B. Differentially abundant proteins (DAPs) found in lemon and orange plants in response to *Trioza erytreae* infestation as compared to the respective controls.

Table C. Loading values of the differentially abundant proteins (DAPs) related to the first component of the principal component analysis (PCA).

Table D. KEGG pathway enrichment analysis for the response of ‘Eureka’ lemon and ‘Valencia’ SwO to *Trioza erytreae*.
