# Supplementary material for: Proteomic analysis may explain differences in Citrus × limon and Citrus × sinensis susceptibility to Trioza erytreae
Source: Plant Signal Behav. 2026 Feb 18;21(1):2632509. doi: 10.1080/15592324.2026.2632509 (PMC12928643; doi:10.1080/15592324.2026.2632509)
Supplement: Figure_B_Representation of the enriched Protein Processing in the Endoplasmic reticulum pathway of EurekaLemonInf and ValenciaSwOInf plants.docx [file KPSB_A_2632509_SM5832.docx]

##
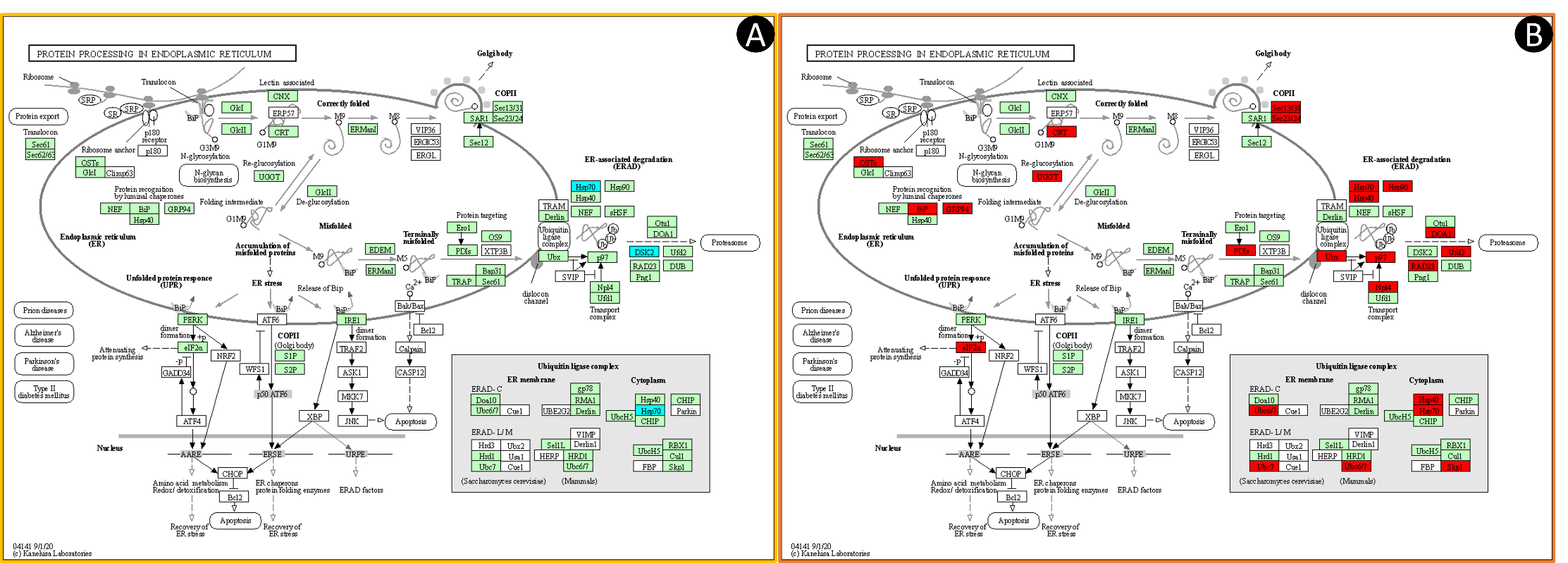


Figure B Representation of the up and downregulated proteins in the “Protein processing in endoplasmatic reticulum” pathway in response to *Trioza erytreae*. Red highlighted forms represent the upregulated proteins and blue highlighted proteins represent the downregulated proteins A - Represents the regulation in the ‘Eureka’ lemon plants (EurekaLemonInf *vs* EurekaLemonCon) comparison. B – Represents the regulation in the ‘Valencia’ sweet orange (SwO) (ValenciaSwOInf *vs* ValenciaSwoCon) comparison.
