# Supplementary material for: Proteomic analysis may explain differences in Citrus × limon and Citrus × sinensis susceptibility to Trioza erytreae
Source: Plant Signal Behav. 2026 Feb 18;21(1):2632509. doi: 10.1080/15592324.2026.2632509 (PMC12928643; doi:10.1080/15592324.2026.2632509)
Supplement: Figure_C_Representation of the enriched pathways of PlantPathogen Interactions Glyoxylate and dicarboxylate metabolism.docx [file KPSB_A_2632509_SM5830.docx]

##
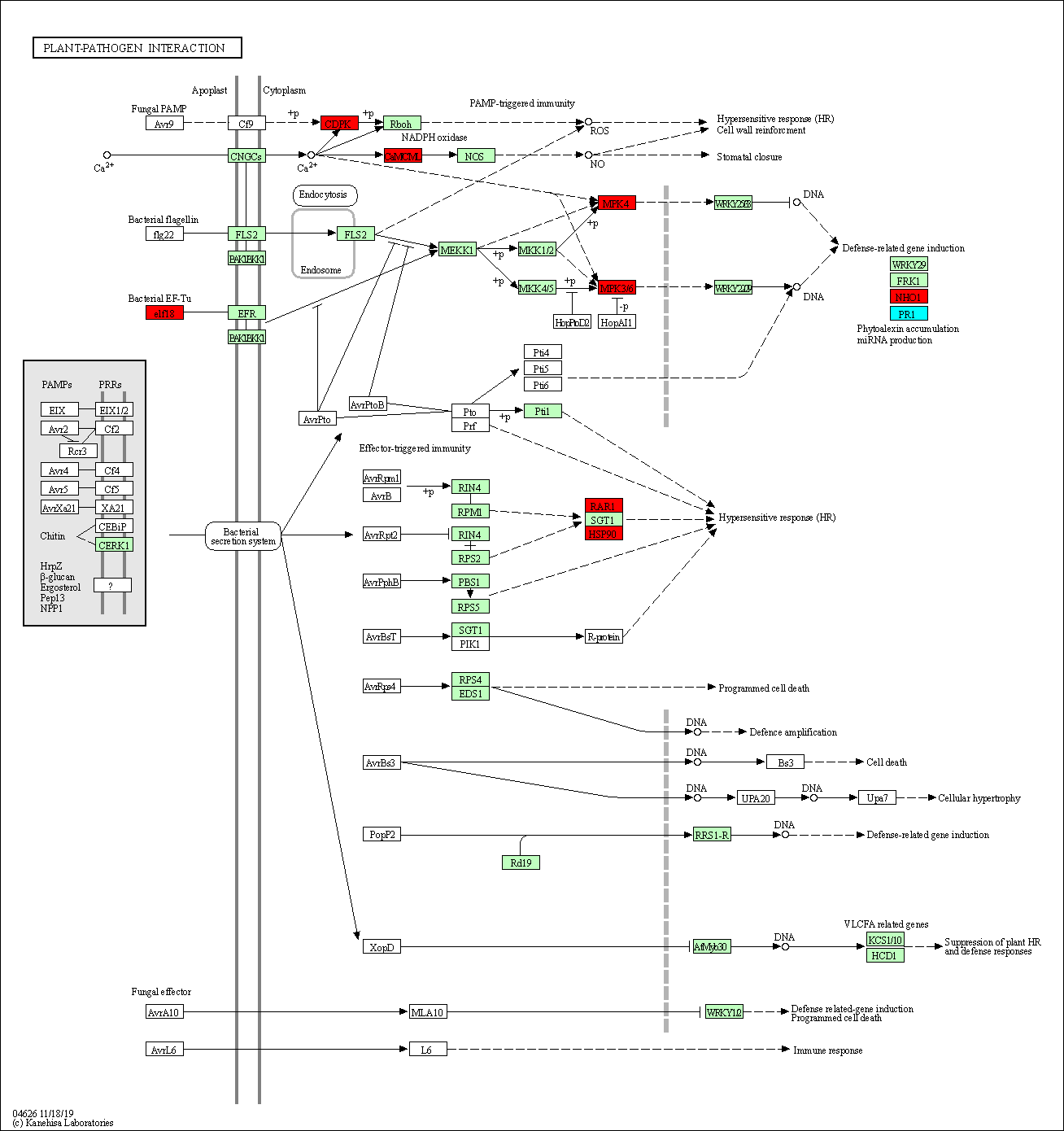

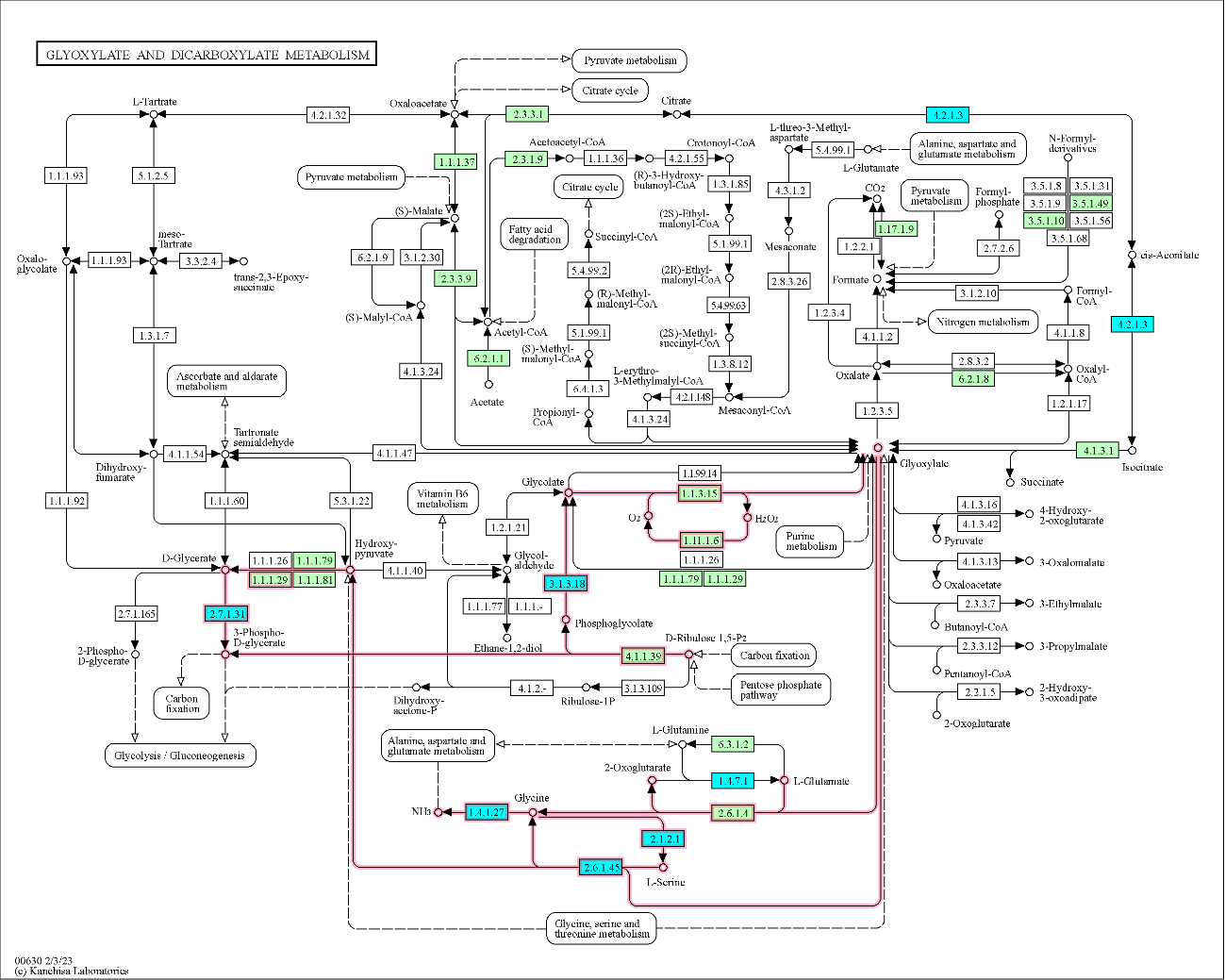

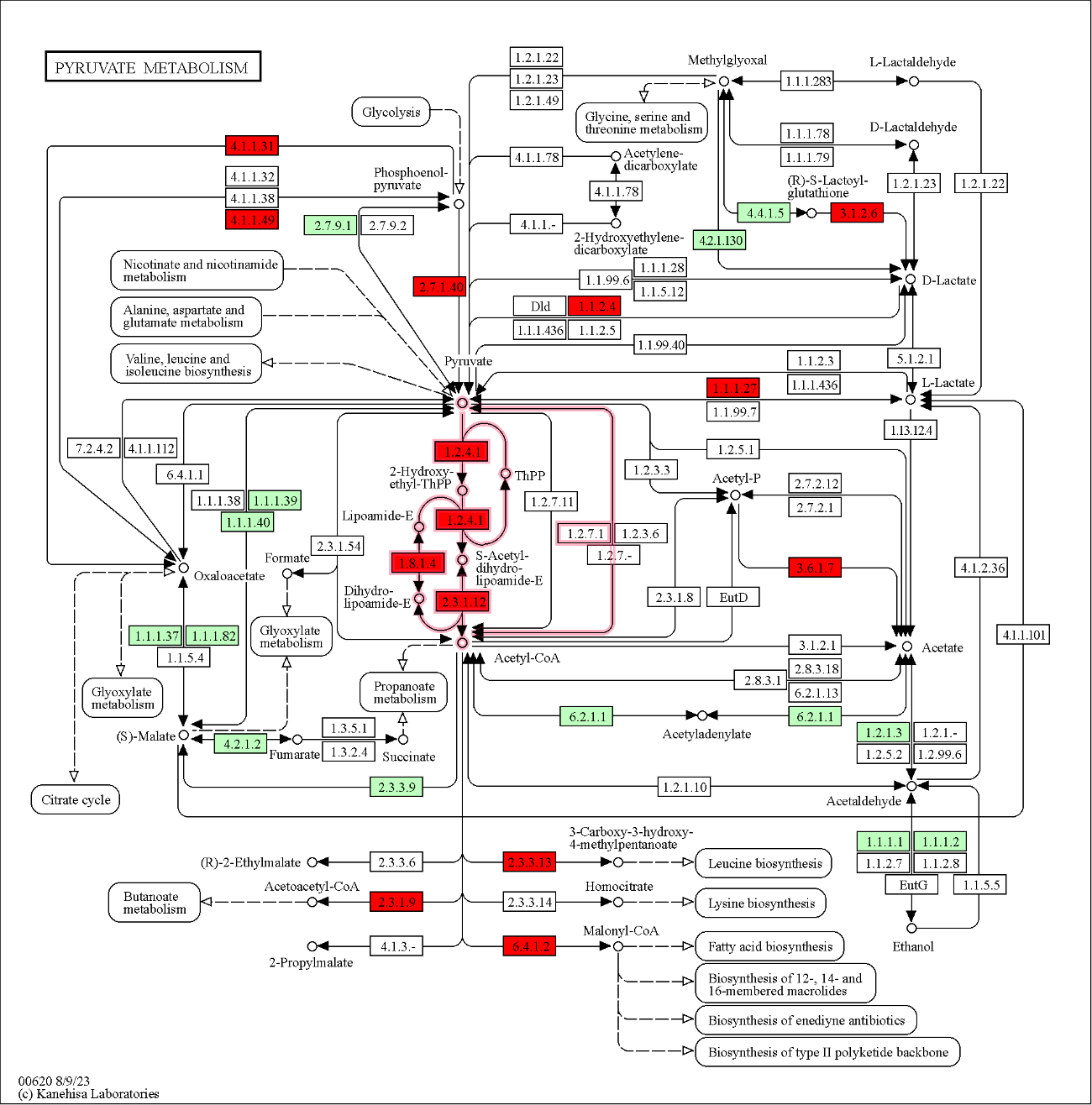

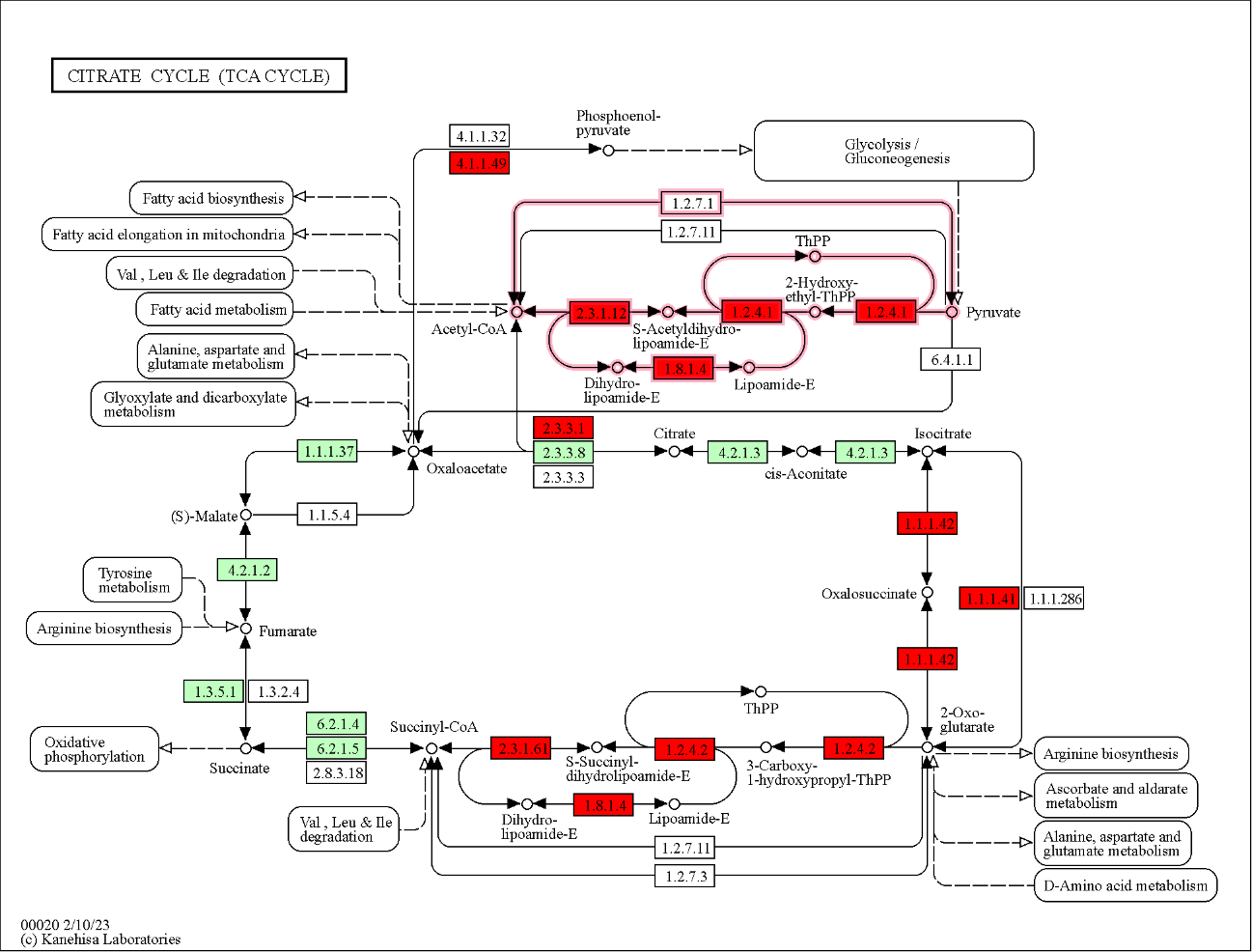


## 1

## 2

## 3

## 4

Figure C Representation of the enriched pathways that resulted from ‘Valencia’ sweet orange (SwO) in response to *Trioza erytreae*, (ValenciaSwOInf *vs* ValenciaSwOCon) comparison. Red highlighted forms represent the upregulated proteins and blue highlighted proteins represent the downregulated proteins. 1: “Plant-pathogen interaction” pathway; 2: “Glyoxylate and dicarboxylate metabolism” pathway, with pink highlighted arrows representing the “photorespiration” module; 3: “Pyruvate metabolism” pathway with the pink highlighted arrows representing the “pyruvate oxidation” module; 4: “Citrate cycle (TCA cycle)” pathway with the pink highlighted arrows representing the “pyruvate oxidation” module.
