# Supplementary material for: Proteomic analysis may explain differences in Citrus × limon and Citrus × sinensis susceptibility to Trioza erytreae
Source: Plant Signal Behav. 2026 Feb 18;21(1):2632509. doi: 10.1080/15592324.2026.2632509 (PMC12928643; doi:10.1080/15592324.2026.2632509)
Supplement: Figure_A_SDS PAGE Gel images of the EurekaLemonInf EurekaLemonCon ValenciaSwOInf and ValenciaSwOCon enriched vascular sap proteome profiles.docx [file KPSB_A_2632509_SM5829.docx]

##
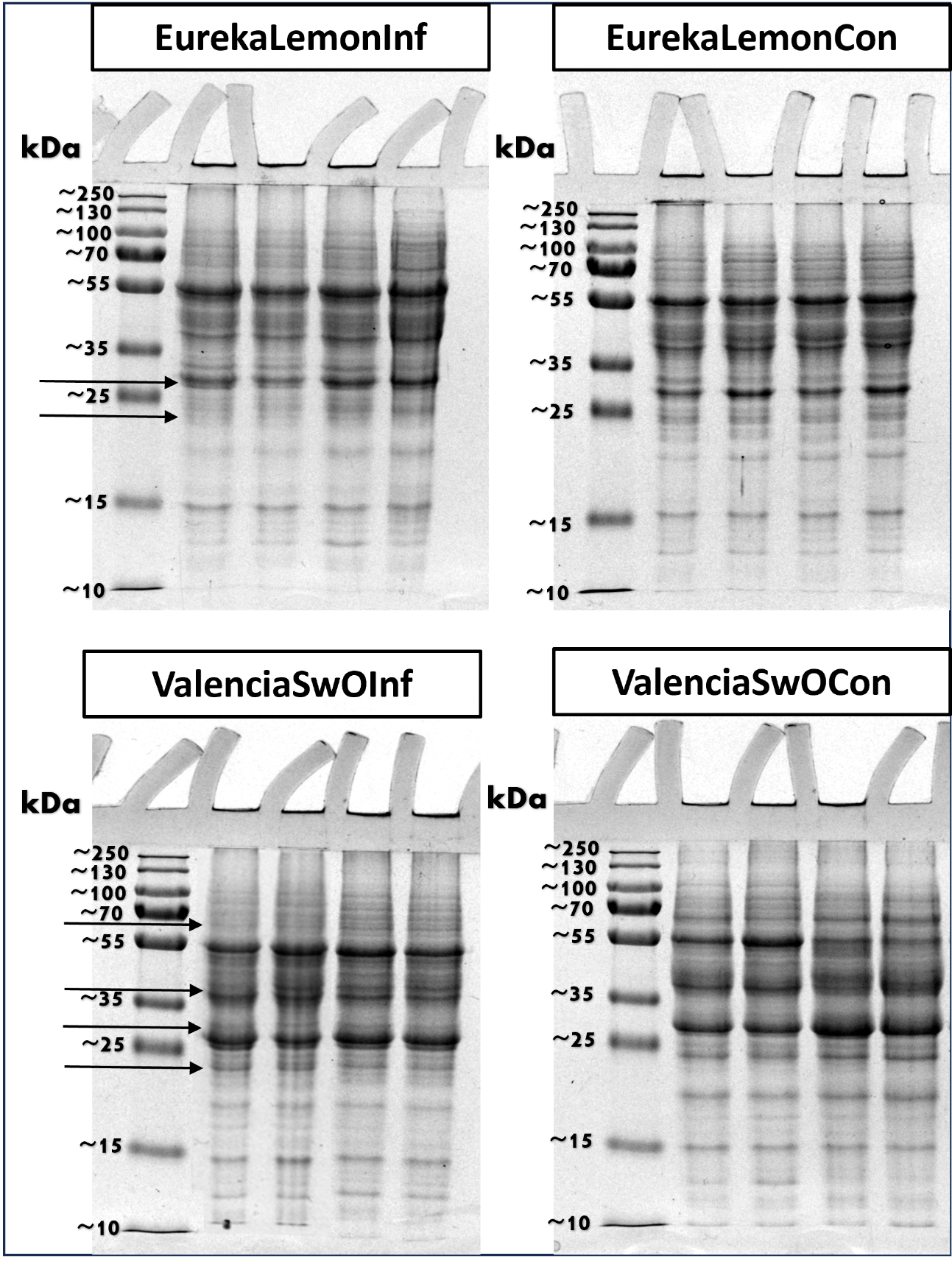


Figure A Protein profile of expressed proteins in the enriched vascular sap of lemon and orange plants in control and infested condition, analysed by SDS-Page (12 %), one gel per condition. The gels named “EurekaLemonCon” and “EurekaLemonInf” represent 4 vascular samples of control and infested ‘Eureka’ lemon plants, respectively. The gels named “ValenciaSwOCon” and “ValenciaSwOInf” represents the 4 vascular samples of control and infested ‘Valencia’ sweet orange (SwO) plants, respectively. Thirty μg protein extracts were run in each well. Arrows highlight the areas with visible differences between infested and control profiles.
